# Blanket antimicrobial resistance gene database with structural information, BOARDS, provides insights on historical landscape of resistance prevalence and effects of mutations in enzyme structure

**DOI:** 10.1128/msystems.00943-23

**Published:** 2023-12-12

**Authors:** Seyoung Ko, Jaehyung Kim, Jaewon Lim, Sang-Mok Lee, Joon Young Park, Jihoon Woo, Zoe K. Scott-Nevros, Jong R. Kim, Hyunjin Yoon, Donghyuk Kim

**Affiliations:** 1 School of Energy and Chemical Engineering, Ulsan National Institute of Science and Technology (UNIST), Ulsan, South Korea; 2 School of Life Sciences, Ulsan National Institute of Science and Technology (UNIST), Ulsan, South Korea; 3 School of Engineering and Digital Sciences, Nazarbayev University, Astan, Kazakhstan; 4 Department of Molecular Science and Technology, Ajou University, Suwon, South Korea; University of Illinois at Chicago, Chicago, Illinois, USA

**Keywords:** antimicrobial resistance, database, analysis pipeline, WGS analysis, predicted protein structures

## Abstract

Antimicrobial resistance (AMR) in pathogenic bacteria poses a significant threat to public health, yet there is still a need for development in the tools to deeply understand AMR genes based on genetic or structural information. In this study, we present an interactive web database named Blanket Overarching Antimicrobial-Resistance gene Database with Structural information (BOARDS,https://sbml.unist.ac.kr/), a database that comprehensively includes 3,943 reported AMR gene information for 1,997 extended spectrum beta-lactamase (ESBL) and 1,946 other genes as well as a total of 27,395 predicted protein structures. These structures, which include both wild-type AMR genes and their mutants, were derived from 80,094 publicly available whole-genome sequences. In addition, we developed the rapid analysis and detection tool of antimicrobial-resistance (RADAR), a one-stop analysis pipeline to detect AMR genes across whole-genome sequencing (WGSs). By integrating BOARDS and RADAR, the AMR prevalence landscape for eight multi-drug resistant pathogens was reconstructed, leading to unexpected findings such as the pre-existence of the MCR genes before their official reports. Enzymatic structure prediction-based analysis revealed that the occurrence of mutations found in some ESBL genes was found to be closely related to the binding affinities with their antibiotic substrates. Overall, BOARDS can play a significant role in performing in-depth analysis on AMR.

**IMPORTANCE:**

While the increasing antibiotic resistance (AMR) in pathogen has been a burden on public health, effective tools for deep understanding of AMR based on genetic or structural information remain limited. In this study, a blanket overarching antimicrobial-resistance gene database with structure information (BOARDS)—a web-based database that comprehensively collected AMR gene data with predictive protein structural information was constructed. Additionally, we report the development of a RADAR pipeline that can analyze whole-genome sequences as well. BOARDS, which includes sequence and structural information, has shown the historical landscape and prevalence of the AMR genes and can provide insight into single-nucleotide polymorphism effects on antibiotic degrading enzymes within protein structures.

## INTRODUCTION

The abuse and misuse of antibiotics have accelerated the emergence of antimicrobial resistance (AMR) in bacteria, leading to multidrug-resistant strains that pose a serious public health threat (1–4). It is important to monitor the trends in AMR gene prevalence to provide the information necessary to limit the spread of these antibiotic-resistant bacterial strains. In particular, an overuse of multiple antibiotics might provide an opportunity for the occurrence of pan-antibiotic resistant infections with ESKAPE pathogens (*Enterococcus faecium*, *Staphylococcus aureus*, *Klebsiella pneumoniae*, *Acinetobacter baumannii*, *Pseudomonas aeruginosa*, and *Enterobacter* species). The ESKAPE pathogens, the major cause of medical infection globally (5), have been under intensive surveillance by the global antimicrobial resistance surveillance system, which was launched in 2015 (6); however, considering their overall mortality and impact, attentive studies are still required. Amidst this context, carbapenems and colistin play a crucial role clinically and from a public health perspective as the last-resort antibiotics against multi-drug-resistant infections (7–11). However, concurrent resistance to these antibiotics not only leads to a significant reduction in available treatment options (10, 12–16) but also is associated with an increased resistance in ESKAPE pathogens, known for their antibiotic evading abilities (17–19). Thus, the need for focused research in this domain is highlighted. Meanwhile, due to progress in next-generation sequencing (NGS) technology (20, 21), whole-genome sequencing (WGS) data and metagenome data have become a valuable resource for screening and evaluating AMR genes. In the field of predicting algorithms of the AMR genes, multiple AMR prediction algorithms have been developed to analyze WGS data (22–24) or metagenome data to predict the existence and characteristics of AMR genes (25–27).

Despite multiple databases contributing to AMR surveillance, the need for structural information remains crucial in understanding the emergence and spread of resistance (28, 29). Protein structure-based functional analysis is essential for uncovering how point mutations impact ligand-protein interactions, including the binding of antibiotics (30) and their effects on vital cellular processes. Thus, improving methods for monitoring point mutations is crucial to comprehend AMR gene spread and enhance understanding of protein function and mechanisms (31, 32). Recent developments in deep learning-based prediction pipelines allow for the utilization of accurate protein structures without experimental demonstration (33–37). The efforts to create enriched resources for various fields of life sciences and structural biology resulted in the construction of multiple databases with protein structures (38) such as UniProt (39), PDB (40, 41), and AlphaFold DB (42). However, the structural information is expected to facilitate in-depth analysis of antibiotic resistance; there is no specific database focusing on AMR genes yet.

Considering these necessities, the blanket overarching antimicrobial-resistance gene database with structure investigation (BOARDS) database, a blanket database including structural information for AMR genes was constructed by multiple sources—database consolidation, literature reviews, and the utilization of protein structure prediction pipelines. The BOARDS database provides genetic information for AMR and their frequently occurring point mutations. To demonstrate the utility of the BOARDS database for in-depth analysis of AMR genes, a massive amount of WGS data including major antimicrobial-resistant pathogens was analyzed using the self-designed pipeline, RADAR. Additionally, the BOARDS database strengthens the basis of extensive AMR research by providing predictive structural information of all included genetic data. Access to the BOARDS database is presented through a web interface (available at https://sbml.unist.ac.kr/). The application of multiple analyses using the BOARDS database illustrates the potential of a novel approach that combines bioinformatics and structural biology to understand antibiotic resistance.

## MATERIALS AND METHODS

### The AMR gene database consolidation

Data on antimicrobial resistance genes were collected from various sources. This process involved a comprehensive and iterative search in scientific literature through PubMed and Google Scholar using keyword such as “novel antibiotic resistance gene,” “AMR gene,” “ESBL,” “carbapenem resistance,” “colistin resistance gene,” “vancomycin resistance gene,” “methicillin-resistance gene,” “erythromycin resistance gene,” and “tetracycline resistance gene.” Subsequently, papers that explicitly mentioned gene names were selected for further examination in the National Center for Biotechnology Information (NCBI) Nucleotide database to obtain the corresponding gene data. Through this process, 890 unique antimicrobial resistance genes were obtained, reflecting the latest trends of antimicrobial resistance. In addition to this literature review, databases such as the Comprehensive Antibiotic Resistance Database (CARD v3.2.1) and the Lahey database were also utilized as sources. CARD provides published sequences, but since the Lahey database does not provide sequences, the accession number registered in NCBI was used to collect the sequences. We finally consolidated an antimicrobial resistance gene database using the collected accession number. These sequences were curated for Open Reading Frame validation before being added to the consolidated antimicrobial resistance gene database. The database consists of a single FASTA file including the gene sequence, and each gene also describes the resources collected and is abbreviated as follows: antibiotic resistance ontology number, CARD; LAHEY, Lahey database; literature, published paper review.

### The prediction of protein structure information

The structure information of all AMR genes included in the BOARDS database was predicted by the AlphaFold2 pipeline to provide a predicted protein structure. In particular, for the single-nucleotide polymorphism (SNP) model provided by the BOARDS database, the protein structure was predicted through AlphaFold2 (34) and RoseTTAFold pipeline (35). The structure prediction pipeline such as AlphaFold2 and RoseTTAFold estimates on a scale of 0–100 for a per-residue confidence score called pLDDT (predicted local distance difference test) for the prediction model. This score, which measures the distance difference between the predicted and actual positions of each protein amino acid residue, has served as an important benchmark for assessing the accuracy of protein structure prediction in prior research (34, 35). Thus, BOARDS database provides the predicted protein structures that are corresponding to the top five based on the average pLDDT of the model. The pLDDT scores were interpreted as follows (34): >90: high accuracy; 70–90: generally good accuracy; 50–70: low confidence; <50: disordered or unstructured.

### The RADAR pipeline development

The Rapid Analysis and Detection tool of Antimicrobial-Resistance (RADAR) was developed as a Linux-based pipeline for AMR gene detection by flexibly referring not only the BOARDS but also other databases. It integrates BLAST and visualization software for AMR gene detection in WGS data. The analysis workflow of the RADAR is as follows. First, prodigal software annotates the genome sequence to identify genetic elements. Second, RADAR optimizes AMR gene detection by referring to the BOARDS database as default and performs rapid local alignment tool (BLAST) searches through Usearch software (43). Third, BLASTp results which focus on total hits and >90% protein similarity and Circos software (44) were used to visualize AMR gene distribution in WGS data. RADAR not only runs on local computers but also supports Google Colab for easy module installation and analysis sharing and utilizes library-form analysis scripts. RADAR packages are available on GitHub (https://github.com/SBML-Kimlab/radar).

To evaluate the detection performance of the RADAR pipeline, a benchmark comparison was conducted using independent WGS data samples against several other AMR detection algorithms [ResFinder (45), AMRFinder (24), RGI (23)]. The AMR detection algorithms chosen for this comparison met the following criteria: (i) a Linux-based pipeline and (ii) capability to support AMR and SNP detection in WGS data. The selection and size of the test data set for the evaluation of the RADAR pipeline were determined based on several considerations. In order to accurately capture the AMR patterns in WGS data and ensure a meaningful performance evaluation, data sets with a high assembly level were selected for testing. Furthermore, WGS data samples from eight major antibiotic-resistant strains were selected using random number generation mechanism to ensure a comprehensive representation of resistance traits. The data set was progressively expanded to include 4,000 sequences for a more thorough evaluation.

### The WGS-based comprehensive analysis

The whole-genome sequence of the eight pathogenic strains was used for RefSeq data from NCBI, and the WGS data that were released until October 2021 was downloaded. The WGS analysis was performed locally developed RADAR, pan-resistome was reconstructed in two ways, and all reconstructing processes were based on WGS analysis: (i) for the Extended Spectrum Beta-Lactamase (ESBL) gene distribution according to strain, a matrix was constructed by counting the detected ESBL hits. The resistome was reconstructed by transforming this matrix to a Jaccard distance matrix and then using principal coordinate analysis to visualize the dissimilarities among samples. (ii) For the resistome for subclass-specific distribution involved creating matrices for each subclass of AMR genes in pathogenic bacteria, followed by normalization (log 10 scale) and heatmap visualization. To investigate newly emerged AMR subclasses epidemiologically, web crawling was performed to collect geographical locations and collection date information and to determine their prevalence and the pre-existence of AMR genes. The procedure for web crawling to collect epidemiological information was conducted as follows. First, GenBank files were searched using the gene ID identified through WGS analysis as the keyword. The API provided by the NCBI Entrez database was utilized to search for the GenBank files of all identified genes. The DBLINK information was then used to identify the corresponding BioSample IDs from the obtained GenBank files. Subsequently, a web crawling process was conducted using these BioSample IDs as keywords. Information such as host, collection data, isolation source, and geographical location was extracted from the attribute column of the BioSample page accessed through this web crawling procedure. Additionally, co-occurrence analysis determined the number of detections of newly emerged AMR genes and confirmed their co-occurrence based on counting results.

### The SNP model adoption

The SNP models from the BOARDS database were determined based on total hits in WGS analysis. In particular, the BOARDS database contains information on frequently occurring point mutations. The process for adopting the SNP models was performed as follows: (i) counting the WGS-based total hits for each AMR gene in the BOARDS database, (ii) calculating the cumulative frequency of the top 1% hits relative to the total hits to identify frequently occurring point mutations, (iii) selecting hits that covered over 50% of the cumulative frequency and performing multiple sequence alignment (MSA) with their reference sequence, and (iv) only non-synonymous SNPs found in the MSA results were adopted as SNP models. The cumulative frequency was applied to explicitly indicate the selected hits through the rank. Thus, the adopted SNP models explained point mutations occurring within the selective hits, which accounted for more than 50% of the total hits.

### The electrostatic potential calculation for SNP effects analysis

The electrostatic (ES) potentials of the model supported by the BOARDS database were calculated using the APBS software. The four models were aligned to set identical residue coordinates before the ES potential calculation. The calculation used default grid point and boundary conditions, with a temperature of 298.15 K (25℃) and no added ions for zero net ionic concentration. The global electrostatic map of each model was visualized in PyMOL, with the biomolecular surface colored from red (−5 kT/e) to blue (+5 kT/e) based on the electrostatic potentials. For calculating atomic ES potentials of each residue, a dx file with scalar fields representing the ES potentials at grid points was used, interpolated relative to each residue coordinates. The atomic ES potential calculation process of ligand was conducted after docking simulation between each of the models and cefotaxime. The calculated atomic ES potentials were used to compare ES differentials caused by point mutations at ligand atoms, and the arithmetic mean of calculated atomic ES potentials was used to compare ES differentials within mutation positions.

### The molecular docking simulation for SNP effects analysis

Docking simulations were conducted to assess the binding affinity between models from BOARDS and the antibiotic cefotaxime. GNINA software, which utilizes convolutional neural networks, was used for scoring. The overall docking process involved global docking and re-docking for each model and the antibiotic (cefotaxime). Global docking parameters included exhaustiveness of search of 256, the number of top poses saved in each Monte Carlo chain of 1000, the number of binding modes to generate of 100, and the amount of buffer space to add to auto-generated box of 4. The ligand with the highest pose score at the active site was selected as the re-docking candidate. Re-docking simulation parameters were exhaustiveness of search of 64, the number of top poses saved in each Monte Carlo chain of 200, the number of binding modes to generate of 20, and amount of buffer space to add to auto-generated box of 4. Redocking was performed 10 times, and the top 3 candidates from each simulation were used for statistical analysis.

### The statistical analysis after independent molecular docking simulation

A total of 30 adopted sampling outputs from the redocking simulations were subjected to statistical analysis. One-way analysis of variance was applied to evaluate the significance of the binding affinity differential relative to each model. Significant differences were observed, and the Tukey’s HSD test was conducted for multiple comparisons. Differences with *P*-value <0.05 were considered statistically significant. Specifically, the mutant models predicted by RoseTTAFold in the BOARDS database were subjected to a *t*-test for evaluating the significance of the binding affinity differentials between each model, but the statistical test with its corresponding models showed that insignificant difference.

## RESULTS

### Construction of a consolidated antimicrobial resistance gene database including predicted protein structure

**Fig 1:**
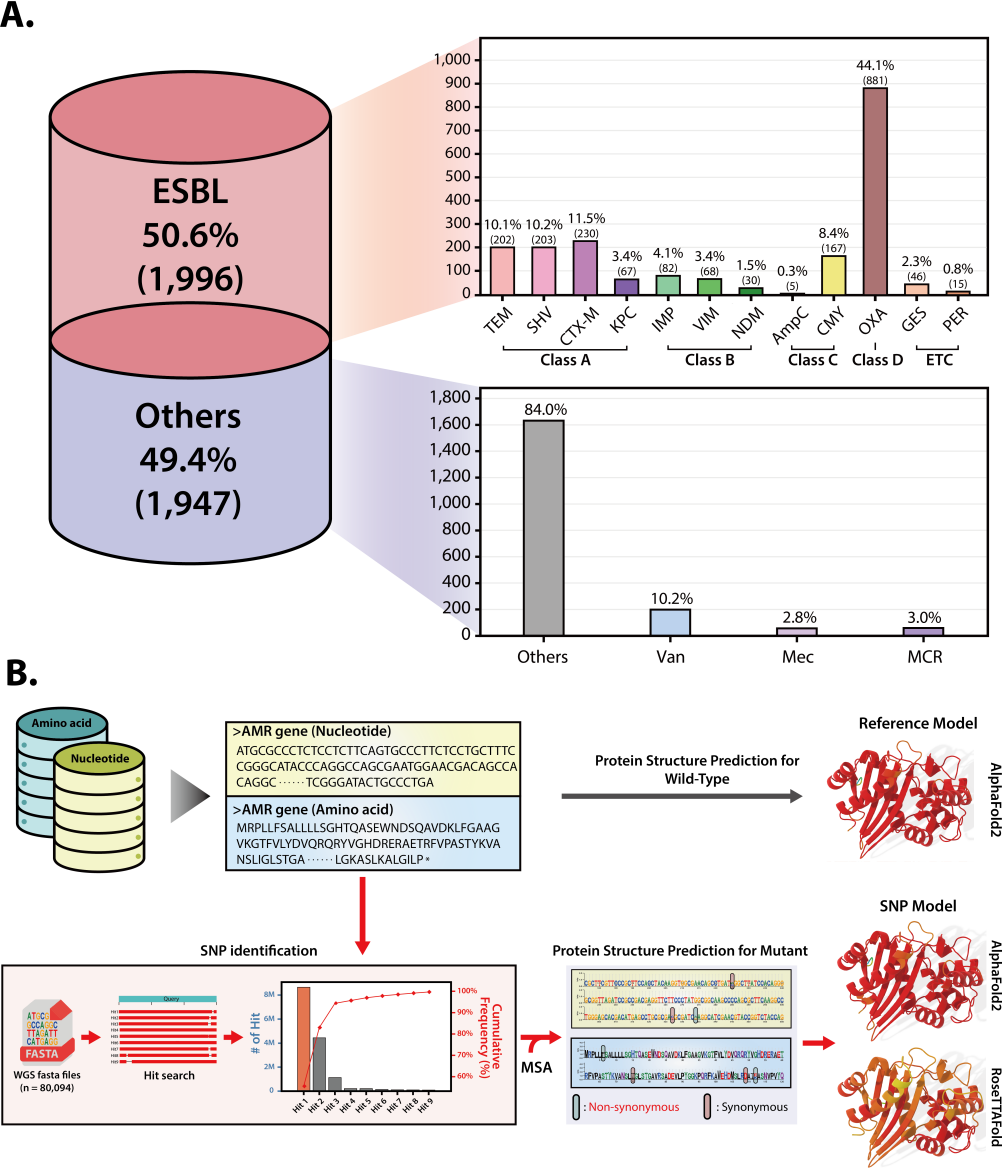
BOARDS database overview. (**A**) BOARDS is a consolidated antimicrobial-resistance gene database that collects AMR gene information from several sources, with more than 50% of the genes representing ESBL (1,997) and the remainder as other AMR genes (1,946). The ESBL genes are categorized based on the Ambler classification: Class A includes genes such as TEM (Temoneira), CTX-M (Cefotaximase-Munich), SHV (sulfhydryl variable), and KPC (*Klebsiella pneumoniae* carbapenemase); Class B comprises VIM (Verona integron-encoded metallo-β-lactamase), IMP (imipenemase), and NDM (New Delhi metallo-β-lactamase); Class C contains CMY (CMY-type beta lactamase) and AmpC (AmpC β-lactamases); Class D features the OXA group (oxacillinase); and Class ETC encompasses GES (Guiana extended spectrum) and PER (*Pseudomonas* extended resistance). Additionally, other AMR genes include Van (vancomycin resistance gene), Mec (methicillin resistance gene), and MCR (mobilized colistin resistance). Genes not covered in the above categories are labeled as others. (**B**) BOARDS provides predictive protein structures for reference models as well as SNP models. The reference model is the predicted protein structure of all AMR genes included in BOARDS, and the SNP model is the predicted protein structure of the adopted non-synonymous SNP model based on SNP information that occurs frequently in eight major multidrug-resistant pathogens. In the reference model, the protein structure was predicted by AlphaFold2, and in the SNP model, the protein structure was predicted by AlphaFold2 and RoseTTAFold.

The BOARDS database is a blanket collection of broad-spectrum AMR genes that are publicly available through multiple sources: literature review and published databases including CARD (23), and Lahey clinic database (http://www.lahey.org/Studies, accessed on April, 2017) (Fig. S1). In the process of consolidating for the construction of the BOARDS, the three primary criteria considered for evaluating available public databases were the depth of bacterial origin data, update regularity, and clarity of labeling for the offered AMR genes (22, 46–48) (Table S1). The BOARDS includes 3,943 AMR gene data from *Eukaryota* and eight bacterial phyla, with majority found in *Proteobacteria*. When investigating the genus level for *Proteobacteria* reveals frequent isolation of *Acinetobacter* (21.9%), *Klebsiella* (17.8%), and *Escherichia* (15.9%), commonly found in clinical environments (Fig. S2). These data show results consistent with previous studies that hospitals can be a conducive environment for obtaining AMR genes (49). The BOARDS database contains multiple AMR genes with variety of characteristics, including ESBL genes according to Ambler classification (50) (Fig. 1A), as well as emerging AMR genes such as MCR, VAN, and MEC subclasses. As a consolidation of available information on AMR genes, the BOARDS contains a larger number of AMR genes than the previously constructed database such as CARD, released in 2020, which collected comprehensive AMR genes (Fig. S3). Furthermore, as the formatting of AMR gene data in the database are uniformly formatted in text format (FASTA format), facilitating easy searching and modification by users.

One of the highlights of the BOARDS database is that it provides predicted protein structures of AMR genes. The BOARDS database contains predicted protein structures of AMR genes, including mutation models based on frequent SNPs in eight major pathogens (51) (Fig. 1B). The structures predicted by BOARDS have an average pLDDT score of 91.97 and a pTM (predicted TM-score) (52) of 86.02, with 99.48% of the genes having a high confidence score of 70 or higher. For mutant models, the top 1% of AMR gene hits are selected by WGS analysis, and the resulting 768 models are cross-validated for SNP impact by structure prediction using Alphafold2 and RoseTTAFold (53) (Fig. S4). Providing structural information with high confidence for every AMR gene in BOARDS can provide a scaffold for biological hypotheses and foundation for informing biological inquiry.

The BOARDS database serves as a valuable resource for investigating AMR genes in a vast amount of bacterial WGS data. Given the recent developments in NGS technology, the amount of WGS data has been increasing exponentially (54, 55) (Fig. S5). To leverage the resource of BOARDS, the RADAR (rapid analysis and detection tool of antimicrobial-resistance gene), an one-stop analysis pipeline for parallel handling of large-scale WGS data was developed. The RADAR pipeline is available as a cloud-based platform utilizing Google Colab to provide user-friendly access. This enables users to identify the distribution of AMR genes in large amounts of WGS data and to employ basal data for more in-depth analysis. It performs automatic gene annotation, local alignment using BLAST, and genome reconstruction visualization to detect AMR genes in pathogen genomes uploaded by users. The RADAR pipeline visualizes the detection frequency of identified mutations in various hosts by comparing the local alignment results of AMR genes found in the pathogen genome with the SNP model information from BOARDS. The RADAR pipeline not only provides results for the detection of AMR genes and SNPs but also emphasizes user convenience by offering multiple supplementary analysis results (Fig. S6). Notably, in case of a detected SNP matching the SNP model of the BOARDS database, a table containing direct links to both the reference and mutant structures is provided. Moreover, it clusters the detected AMR genes in the pathogen genome with all AMR genes in BOARDS, providing insight into the similarity between the detected AMR genes in the pathogen genome and the AMR genes in BOARDS. Subsequently, RADAR provides a phylogenetic tree of the representative sequences of each AMR subclass cluster and the AMR gene hits detected in the target pathogen genome.

**Fig 2:**
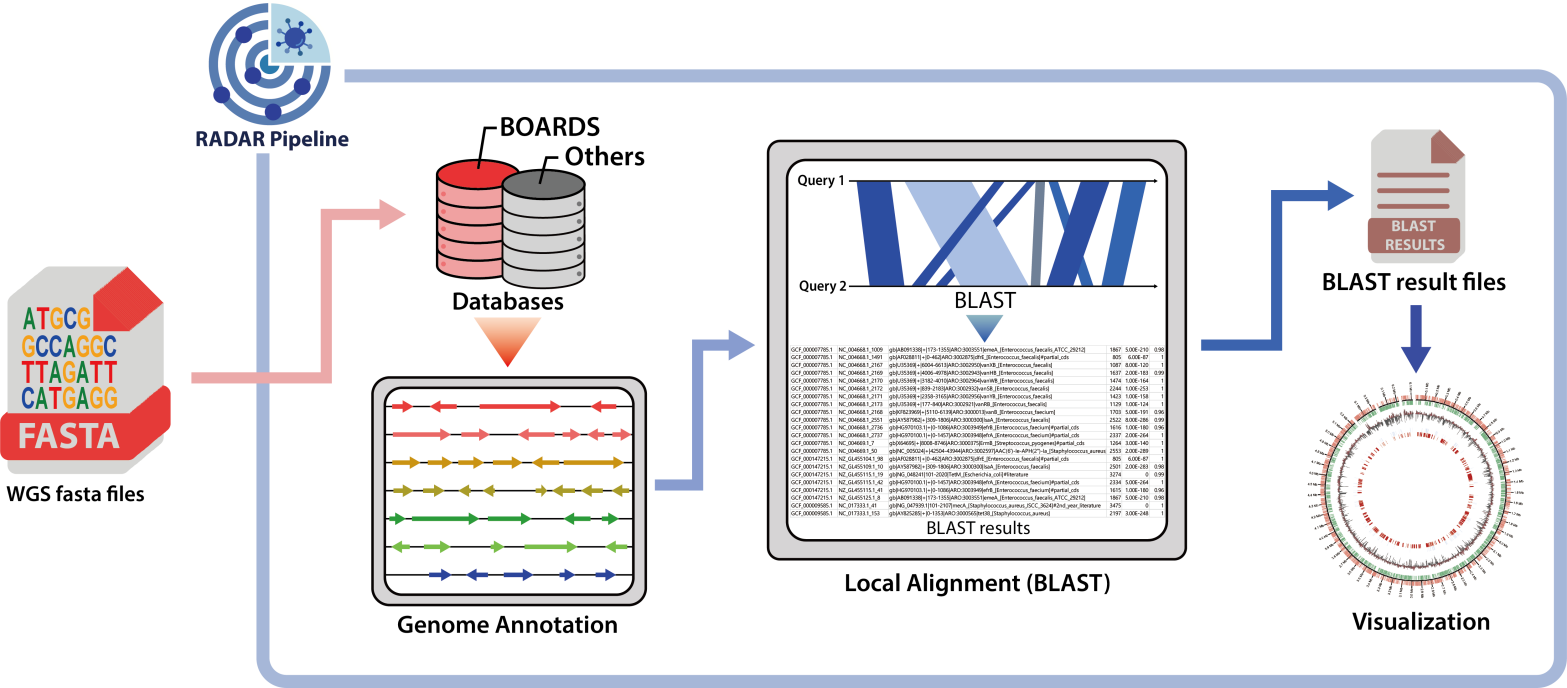
The schematic representation of the RADAR pipeline. The RADAR pipeline is a WGS analysis pipeline that consists of three core infrastructures: annotation, local alignment, and visualization. Moreover, the database for local alignment used in the RADAR pipeline can be configured according to the research purpose, and all processes for WGS data analysis are performed automatically. The RADAR pipeline provides a cloud-based analysis pipeline that utilizes the Google Colab platform to perform analysis with the same performance as a local environment.

The performance of the RADAR pipeline was assessed in various aspects compared to several previous AMR detection algorithms. Three datasets of multiple sizes (40, 400, 4,000 of WGS data) were constructed using randomly selected WGS data from eight major antimicrobial-resistant pathogenic bacteria. As a result of comparison, the RADAR pipeline was found to have comparable AMR detection capabilities to those of the RGI software developed by CARD, and it detected more SNPs compared to other tools that were evaluated (Fig. S7A and B). Additionally, the detection performance for multiple AMR classes was assessed, and the RADAR pipeline displayed high performance in detecting core AMR classes, such as methicillin, macrolide, and aminoglycoside resistance compared to other algorithms (Fig. S7C). Notably, RADAR sets itself apart from other AMR detection tools by offering interoperability with multiple AMR databases (Fig. 2). This enables users to perform extensive analyses of AMR genes using diverse resources, thereby offering a biological insight that goes beyond an independent AMR detection tool. Through its key features, including streamlined operation, cloud-based platform, and compatibility with multiple other databases, RADAR can be presented as a viable alternative to other previous tools (23, 24, 45, 56).

### Web-based BOARDS database server for browsing antimicrobial resistance gene data

**Fig 3:**
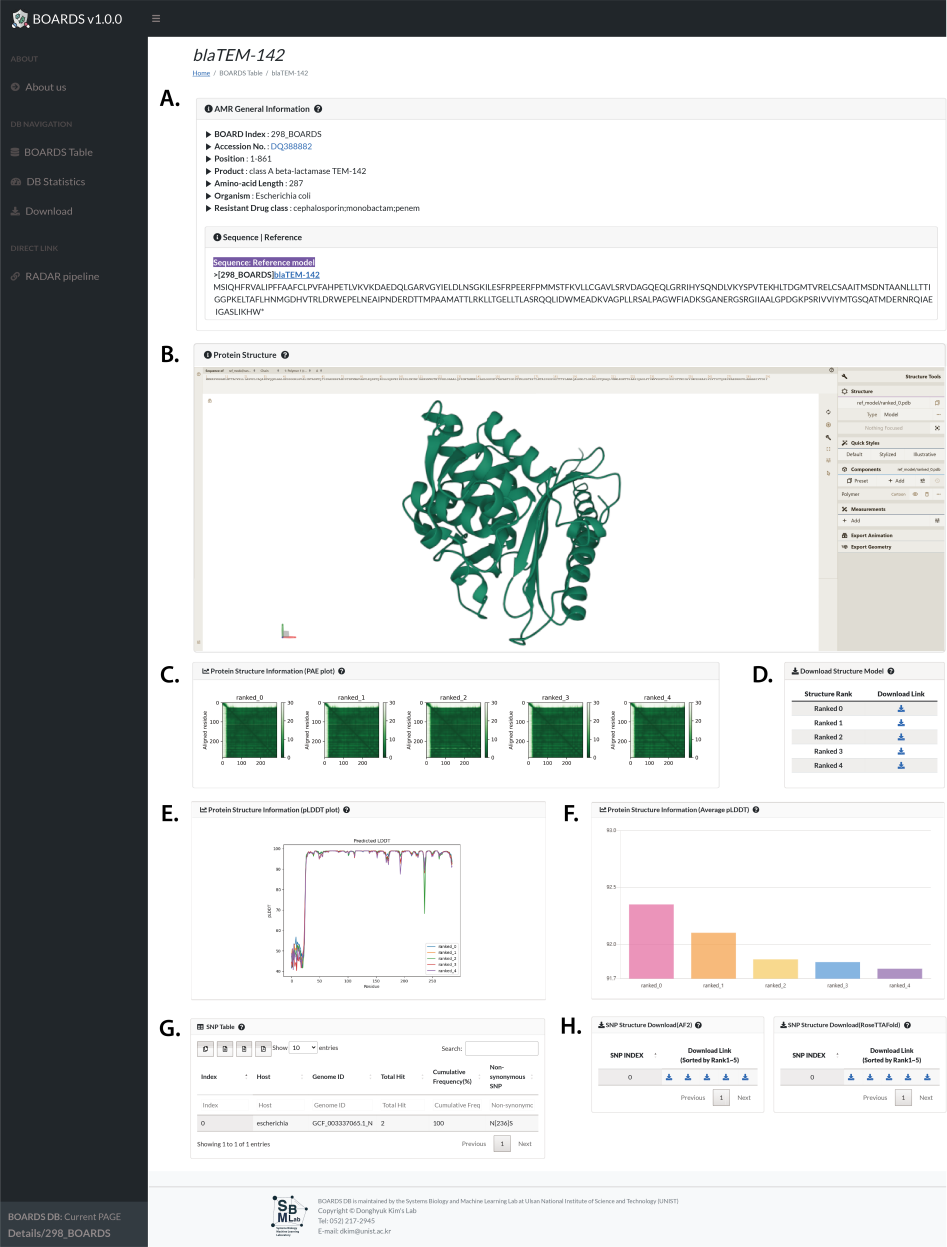
Details webpage for AMR genes in BOARDS database. (**A**) General information about a given AMR gene. (**B**) 3D structural visualization with the highest pLDDT score of the predicted protein structure of the AMR gene. (**C**) PAE plot of the predicted protein structure of AMR gene. (**D**) Download links for the corresponding predicted protein structures. (**E**) pLDDT plot of the predicted protein structure of the AMR gene. (**F**) pLDDT score of the predicted protein structure of the AMR gene. (**G**) SNP table with high frequency identified based on WGS analysis when non-synonymous SNPs were detected. If no non-synonymous SNPs were detected, the message “No data available in table” is displayed. (**H**) Download link for structurally predicted protein structures of the SNPs. The links on the left and right provide access to the protein structures predicted by AlphaFold2 and RoseTTAFold, respectively. If no identical SNPs are found, the message "No Results" is displayed.

To enhance the accessibility of the information on AMR genes and their predicted protein structures, we present a web-based database server for BOARDS (available at https://sbml.unist.ac.kr), which allows users to easily access information on AMR genes and their predicted protein structures. The BOARDS web server includes three main links: (i) About us; (ii) DB NAVIGATION, which contains the three main contents (BOARDS Table, DB statistics, Download); and (iii) a DIRECT LINK to the RADAR pipeline for utilizing BOARDS. The About us page provides an overview of the contents of BOARDS web server. The DB statistics page provides an overview of statistical information on AMR genes, including genetic data and drug class. The BOARDS table provides detailed information on AMR genes, including an index, gene name, accession number, position, product, organism, drug class, and a column to indicate whether a SNP model detects. If non-synonymous SNPs were detected by WGS analysis, the “SNP model” column is labeled as “Detected” and the number of models is shown. Additionally, further details are available using the links in the “Details” and “AMR Gene” columns. Through the link provided in the “Details” column, users can access the webpage containing the predicted protein structure information and SNP information of the corresponding AMR gene and can download the entire protein structure model predicted by various structure prediction pipelines (Fig. 3). In addition, AMR genes with SNP model present the SNP information as a table in the “Details” link and provide download links to protein structures of SNP mutants predicted by AlphaFold2 and RoseTTAFold (Fig. S8). The “DIRECT LINK” allows users to access the GitHub repository where the RADAR pipeline is stored. Clicking on this link, users can access the code and detailed documentation on how to utilize the RADAR pipeline for analysis. The BOARDS website includes descriptions for all content; users can hover over a question mark to pop up a short description of that content.

### Sequence-based analysis of AMR genes in antimicrobial-resistant bacteria using BOARDS

**Fig 4:**
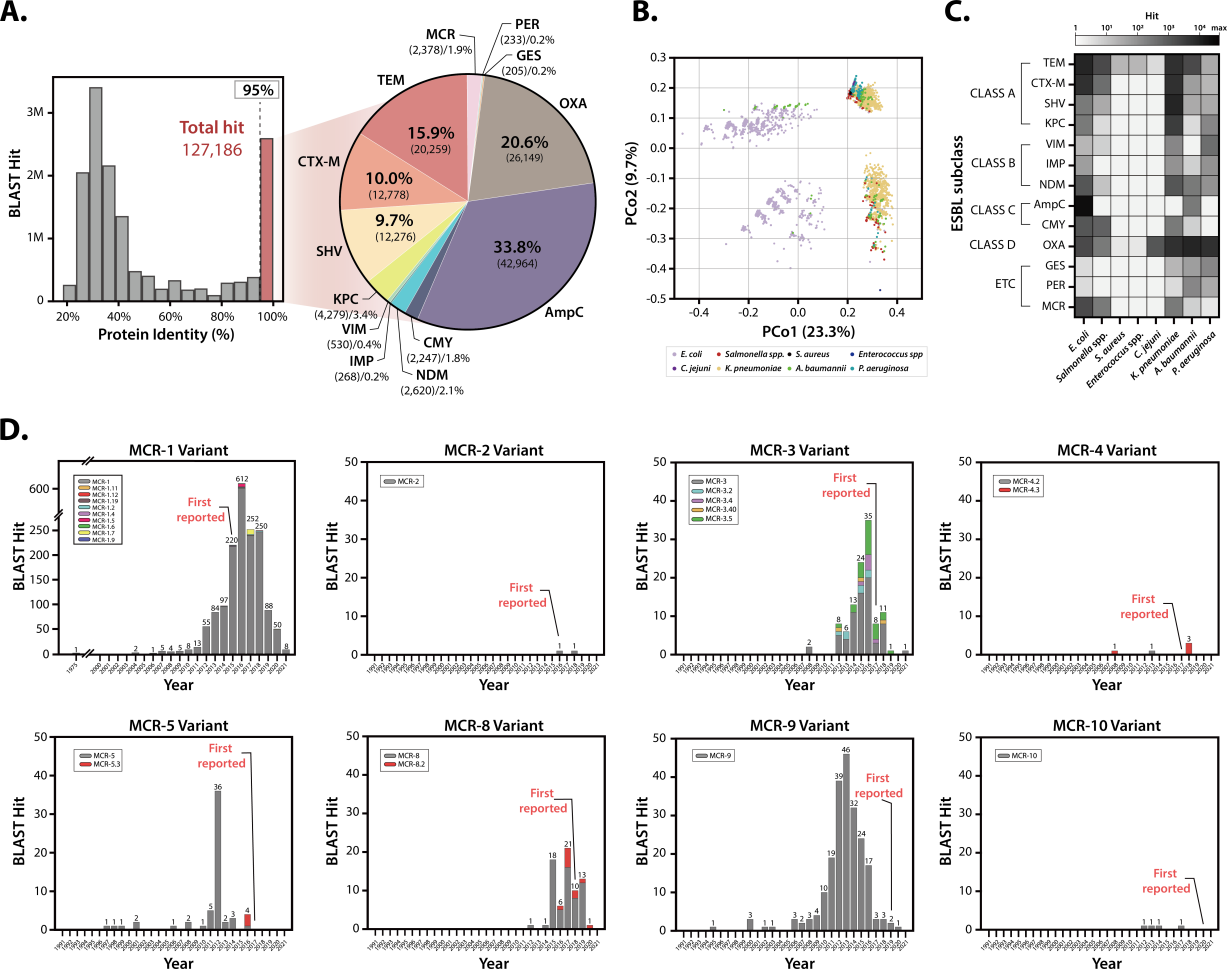
The results of WGS analysis used BOARDS and RADAR pipeline and ESBL gene detection results within eight major pathogen WGS. (**A**) Analysis of eight multidrug-resistant pathogens corresponding to a total of 80,094 WGS confirmed that 127,186 ESBL hits with protein similarity of 95% or more were detected. The most frequently detected ESBL subclasses were AmpC, OXA, TEM, CTX-M, and SHV. (**B**) ESBL distribution status by pathogen type classified through the reconstructed pan-resistome. The reconstructed pan-resistome showed that *E. coli* and *A. baumannii* were globally assorted, and it was confirmed that *K. pneumoniae*, *P. aeruginosa*, and *Salmonella* spp. had a similar ESBL distribution patterns. In addition, it was confirmed that *Enterococcus* spp. and *S. aureus* showed a very similar distribution, and *C. jejuni* showed a consistent distribution pattern detected in the species. (**C**) The distribution overview of the ESBL subclass in each pathogen confirmed through the reconstituted pan-resistome. Through the reconstructed pan-resistome, the dominant ESBL subclass for each strain could be identified, and it was shown that the strain with the most ESBL distribution was *E. coli*. (**D**) The distribution of the reported year of the WGS detected MCR subgroups. In all graphs, the *x*-axis represents the year, and the *y*-axis represents the number of hits in the corresponding MCR subgroup. Additionally, the first reporting year of the corresponding MCR subgroup is indicated in each graph.

In order to validate the effectiveness of the BOARDS database and the developed RADAR, a comprehensive analysis of ESBL occurring within the eight major antimicrobial-resistant pathogens, including ESKAPE, *C. jejuni*, and *Salmonella* species (57), was performed. A total of 14,600,841 hits were detected, with 127,186 cases of ESBL-associated AMR genes selected based on sequence identity above 95% (Fig. 4A; Table S2). In addition, out of 80,084 WGS data, 52,543 genomes had detected AMR genes. The most frequent ESBL subclasses were AmpC, OXA, TEM, CTX-M, and SHV subclasses.

To explore the distribution pattern across major antimicrobial-resistance strains in detail, the pan-resistome was reconstructed through the RADAR according to the following criteria: (i) type of bacteria (Fig. 4B) and (ii) ESBL subclass (Fig. 4C) (Table S3). The distribution patterns based on strains revealed the global spread of *E. coli* and *A. baumannii*. Three bacteria, *K. pneumoniae*, *P. aeruginosa*, and *Salmonella* species had similar distribution patterns, while *C. jejuni* showed consistent distributions within the strains. The overall distribution of *Enterococcus* species closely resembled that of *S. aureus* (Fig. S9). The analysis based on subclass-specific distribution (Fig. S10 to S14) showed the number of ESBL hits in different strains, with *E. coli* having the highest count of 65,224, followed by *K. pneumoniae* (35,489), *A. baumannii* (12,761), *P. aeruginosa* (7,511), *Salmonella* spp. (3,485), *C. jejuni* (1,476), *S. aureus* (17), and *Enterococcus* spp. (15). The dominant subclasses varied among the strains, with TEM being the most prevalent in multiple strains. In summary, the reconstructed pan-resistome revealed that the distribution of ESBL genes in *E. coli* and *A. baumannii* was found to be global, and *E. coli* was confirmed to be the bacteria harboring the most ESBL genes.

Due to the significance of carbapenem and colistin resistance, which are considered the last-resort antibiotics against multi-drug resistant infections, a thorough investigation of their genetic resistance profiles was conducted. Therefore, the epidemiology of two carbapenem-related AMR subclasses (KPC and NDM) and a colistin-related MCR subclass, which are all globally emerging AMR subclasses, was investigated through monitoring the prevalence tendency for each subclass based on the WGS analysis (Table S4). All three subclasses showed an exponential increase in occurrence (Fig. S15A). The KPC subclass had 27 variant alleles, with the largest increase observed in 2013. Regarding the NDM subclass, it had 17 variant alleles, with the highest occurrence in 2019, and some hits were pre-existed before the reported year. In perspective of the geographical source of the pre-existed cases, the NDM subclass were mainly isolated in India (52%), the United Kingdom (42%), and Germany (8%), respectively. The MCR subclass had 24 variant alleles, with the highest occurrence in 2016, and 530 hits were pre-existing genes before 2015 when the subclass was first reported. Notably, pre-existing genes were identified prior to the time each subgroup was first reported in MCR subgroup except for the MCR-2 subgroup (Fig. 4D). An epidemiological investigation showed that pre-existing genes in MCR subclass had been isolated from 39 countries, the top five of which were China, Japan, USA, Spain, and Paraguay. In particular, the first isolated hit was found in 1975, which suggests that the colistin-resistance gene was discovered 16 years after clinical use of colistin (58). Consequently, epidemiological analysis via BOARDS and the RADAR not only showed the prevalence of multiple admixture events within the three newly emerged AMR subclasses but also suggested the pre-existence of several MCR subclass alleles through additional manual tracing of WGS information.

Based on the epidemiologic result that KPC, NDM, and MCR subclasses were found only in *E. coli*, *Salmonella* spp., *K. pneumoniae*, *A. baumannii*, and *P. aeruginosa*, the co-occurrence of carbapenem and colistin resistance genes was analyzed as well (Fig. S15B; Table S5). Analysis showed that the number of hits for KPC, NDM, and MCR subclasses were 4,161; 2,563; and 2,281, respectively. Multiple subclass detection occurred in certain strains, such as a *K. pneumoniae* strain with KPC-1 and KPC-19, and an *E. coli* strain with NDM-5 and NDM-20. In the MCR subclass, the multiple MCR combinations were also identified, with 68 strains possessing 14 MCR combinations. Moreover, multiple co-occurrence patterns were observed (Fig. S15C), while some strains contained all three subclasses simultaneously. The co-occurrence ratio between subclasses varied across species, with the MCR subclass more frequently co-occurring with the NDM subclass. Simultaneous occurrence of multiple subclasses indicates resistance to multiple antibiotics. The detection of strains with simultaneous presence of KPC, NDM, and MCR subclasses suggests frequent co-occurrence of carbapenem and colistin antibiotic resistance genes.

### Structure-based analysis for mutation effect with predicted protein structures in BOARDS

The global spread of AMR genes has been confirmed through WGS analysis, but understanding the diversity of molecular mechanisms due to point mutations remains challenging. To investigate the effects of frequent point mutations, protein structure models of wild-type and mutant variants provided by the BOARDS database were utilized. The focus was on SNPs occurring near the active site of AMR genes, as they play a role in neutralizing antibiotic efficacy. The analysis primarily focused on beta-lactamase, known for its resistance to locally used beta-lactam antibiotics. The TEM subclass on ESBL, which was already experimentally characterized the effects of multiple single amino acid substitutions (59), was used to validate the analysis. TEM-1 (WT) and TEM-52 (WT) models were selected for the SNP analysis, along with TEM-142 (WT) and TEM-142 (mutant) models, which have similar mutations (60). Compared to the TEM-1 (WT) model, there were four positions (104, 182, 238, and 263) at which mutations occurred between the other three models. Mutation types for each target model are as follows: TEM-52 (WT): E104K, M182T, and G238S; TEM-142 (WT): E104K, G238N, and T263M, and TEM-142 (mutant): E104K, G238S, and T263M.

**Fig 5:**
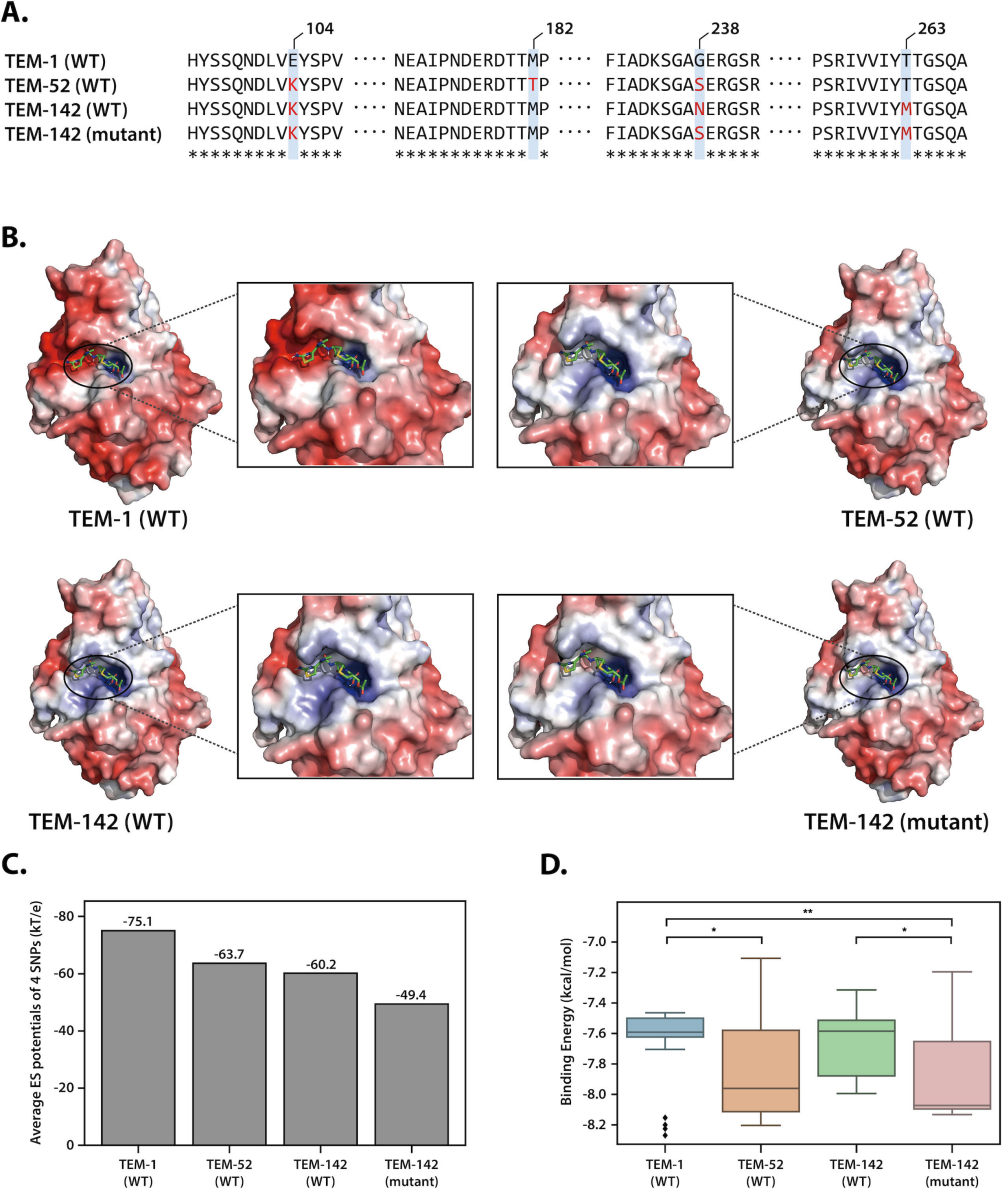
The overview of SNP effects analysis using the predicted protein structure provided by BOARDS. (**A**) The major SNPs occurring in the selected four TEM subclass mutation models. (**B**) The global ES density map of four models [TEM-1 (WT), TEM-52 (WT), TEM-142 (WT), TEM-142 (mutant)] including the major mutants of the TEM subclass (Blue, positive ES potentials; red, negative ES potentials, ranging from −5.0 to 5.0). The enlarged portion is the electrostatic map of the substrate binding site. (**C**) The average of the electrostatic potential of the four mutation positions (104, 182, 238, 263) for each model. (**D**) The binding energy differential between each of the four models and cefotaxime.

The effect of point mutations in AMR genes encoding antibiotic hydrolytic enzymes was investigated through analysis of electrostatic potentials (ES). As a result, TEM-52 (WT), with more SNPs, showed higher total electrostatic energy compared to TEM-1 (WT), as did TEM-142 (mutant) compared to TEM-142 (WT) (Fig. S16). Global electrostatic density maps clearly revealed ES differentials around the active site for the models (Fig. 5B). TEM-52 (WT) had more positive ES potentials, while TEM-142 (mutant) had more negative potentials. In-depth analysis of four positions (104, 182, 235, and 263) showed significant ES differentials based on SNP presence (Table S6). Notably, position 238, with diverse substitutions, had more negative potentials when glycine was substituted (Fig. S17). It was inferred that ES distribution for cefotaxime would be influenced by an E104K mutation, which is the most common in the three models derived from TEM-1 (WT). Moreover, based on the previous study that E104K affects the oxime substituent of cefotaxime (61), an additional analysis was performed to understand the correlation between E104K and ligand ES environment modulation (Fig. S18). Similar to the global ES distribution of cefotaxime, the average ES potential of the atoms corresponding to the oxime group (C24, C25, N14) showed a negative potential (−2.78) for TEM-1 (WT), whereas a positive potential was observed for the other three models. The ES potential for TEM-52 (WT) was calculated to be 1.88, and the ES potentials for TEM-142 (WT) and TEM-142 (mutant) were calculated to be 2.13 and 1.36, respectively (Table S7). The positive potentials could suggest that the presence of the E104K mutation may induce an attraction force on the oxime group in cefotaxime.

Furthermore, independent docking simulations were conducted to assess the binding affinities of cefotaxime with each model, validating the impact of SNPs on antibiotic efficacy (Fig. 5D). The simulations demonstrated that models with mutations showed lower binding energies. Specifically, TEM-52 (WT) demonstrated lower binding energy than TEM-1 (WT), and TEM-142 (mutant) showed lower binding energy compared to TEM-142 (WT) (Table S8). Additional docking simulations were conducted using RoseTTAFold-predicted models of TEM-142 (WT) and TEM-142 (mutant), as the BOARDS database includes mutant models predicted by RoseTTAFold. These simulations confirmed the lower binding energy of the mutant model although it was not statistically significant (Fig. S19). Considering that the ESBL gene-encoding enzyme directly binds to the antibiotic to hydrolyze it, the simulation results suggested that TEM-52 (WT) might hydrolyze cefotaxime more efficiently by binding more strongly than TEM-1 (WT). Moreover, it is possible that TEM-142 (mutant) forms a stronger bond with cefotaxime than TEM-142 (WT) through N238S point mutation according to the docking simulation. These docking simulation results suggested the feasibility of the predicted protein structure within BOARDS database to explore molecular characteristics of antibiotic-degrading enzymes.

## DISCUSSION

The continuous overuse and misuse of antibiotics have led to the transfer of antibiotic-resistance factors to pathogenic bacteria, causing the emergence and rapid spread of AMR genes across multiple species. This phenomenon has been stipulated as a serious threat by the World Health Organization in their global reports (6). However, there is an insufficient number of interconnected databases for in-depth analysis of AMR genes. To address this, the BOARDS database collects comprehensive AMR gene information, including frequently occurring mutations obtained from WGS analysis. This facilitates detailed analysis of SNP effects within AMR genes. Since WGS data provide high-resolution insight into the entire bacterial genome, it is crucial for monitoring AMR genes in major antibiotic-resistant pathogens. Moreover, by utilizing the predicted protein structures of AMR genes available in BOARDS, deeper insights into AMR genes can be achieved. Although multiple algorithms for predicting AMR already exist (22–27, 45–47), RADAR, a cloud-based one-stop pipeline for WGS analysis, has been developed to analyze in conjunction with the BOARDS database. The RADAR pipeline facilitates the detailed and easy screening of homologous AMR genes within large-scale WGS data. RADAR differentiates itself from other AMR detection tools by providing interoperability with multiple AMR databases including BOARDS. This allows users to perform a wide range of analyses on AMR genes using different resources and acquire diverse biological insights. In the comparative evaluation, RADAR was observed to have higher performance in detecting specific resistance classes, especially methicillin, macrolide, and aminoglycoside. However, for other AMR classes, its performance was found to be similar to that of other detection tools. Such comprehensive performance insights not only underscore the potential benefits and scope of RADAR but also serve as a guide for users, enabling them to select and utilize tools such as RADAR with a focus on their research objectives and pertinent AMR classes. While each tool is optimized for its specific purposes, considering the unique capabilities of RADAR, especially its integration with the BOARDS database can be an advantageous choice for researchers aiming for comprehensive AMR gene analyses. It is important to note that the sequence quality of WGS data plays a significant role in the accuracy of analysis results, as the pipeline does not include additional sequence validation steps.

The WGS analysis using the BOARDS database and the RADAR identified prevalent ESBL-associated AMR genes in eight major pathogenic strains. In particular, the reconstructed pan-resistome revealed distribution patterns of ESBL genes among different species, with *E. coli* and *A. baumannii* being widely spread and *E. coli* showing diverse ESBL genes. By reconstructing the pan-resistome, characteristic ESBL subclasses were identified for each bacterium. Meanwhile, the WGS analysis was used for epidemiological analysis as well, showing the prevalence of newly emerging AMR genes including ESBL genes. The AMR genes to be analyzed were the KPC subclass and the NDM subclass, which are dominant carbapenemases, and the MCR subclass, a colistin-resistance gene that has the potential to produce pan-drug-resistant strains by being transferred to CRE (62). Epidemiological investigations have revealed a rapid increase in new AMR gene subclasses, including NDM and MCR, indicating the presence of multiple variant alleles. Notably, the MCR-1 allele was identified as the most pre-existing case within the MCR subclasses (63). Additionally, it was observed that alleles for the most MCR subclasses already existed before they were first reported (64–70). Co-occurrence analysis of the emerged AMR subclasses was conducted, and more importantly, the strains found by the analysis were consistent with the previous studies (71–74). Overall, these results suggest an increasing occurrence of newly emerging AMR subclasses and the co-occurrence of antibiotic resistance among bacterial strains.

The BOARDS database contains predicted protein structures for frequently occurring mutants using AlphaFold2 and RoseTTAFold. Despite concerns about the credibility of these algorithms (53, 75, 76), the SNP analysis conducted in this study suggests that predicted structures in the database may offer insights into the impact of SNPs on antibiotic-degrading enzymes. Comparisons were made between empirically elucidated structures of TEM-1 and TEM-52, as well as TEM-142 (WT) and TEM-142 (mutant). Analysis of electrostatic (ES) density maps revealed clear differences caused by major SNPs. Additionally, the quantitative assessments of local ES potentials and ligand interactions showed that amino acid substitutions influenced the ES environment. In particular, the E104K mutation is likely to strengthen the attraction force to the oxime substituent of cefotaxime, which is supported by the results of previous studies mentioning the effect of E104K on the substituent (61). In the subsequent docking simulations, the SNP models showed lower binding affinities, suggesting stronger binding of cefotaxime to TEM-52 (WT) and TEM-142 (mutant) compared to their respective reference models. These findings indicate that SNPs at the active site could impact the hydrolytic properties of enzyme against third-generation antibiotics though additional verification is needed. Nevertheless, these results of integrated ES and docking simulation analyses demonstrate the potential of protein structure prediction tools in identifying mutation effects.

Due to the recent remarkable development in the field of structure prediction, the AlphaFold Protein Structure Database (42), a gigantic database that predicts the structure of all proteins in the proteome in a batch using nearly every organism with protein sequence data, which is searchable in the UniProt database, has been updated (77). Compared to BOARDS, approximately 93% (3,661/3,943) of AMR gene data can also be found in the AlphaFold Protein Structure Database, while 7% that exist within some strains of a specific organism that AlphaFold Protein Structure Database does not contain, thus highlighting the wide coverage of BOARDS for AMR genes. As the availability of 3D protein structures increases, the use of predicted protein structures in analysis can provide valuable insights into the stereochemical environment of AMR genes. However, there are limitations in the credibility of predicted structures that have not been confirmed by X-ray crystallography or NMR spectroscopy (78, 79). Therefore, BOARDS not only provides the AlphaFold2-oriented structure prediction for mutations in AMR genes identified based on WGS analysis but also provides structural information predicted through RoseTTAFold (35), another representative structure prediction pipeline. In other words, BOARDS stands as more of a mere collection of 3D structures; rather, it embodies a database where the integration of multiple 3D structure prediction models enhances accuracy and reliability, thereby ensuring its robustness as a comprehensive resource for AMR gene information.

The present study showcases the development of the BOARDS database, which has provided insights into the prevalence and influence of AMR genes. Also, the SNP analysis using information of predicted structures demonstrates its potential for understanding AMR genes and expanding into other research areas. While the current release of BOARDS, with its rich content, user-friendly interface, synchronized gene nomenclature, and downloadable data, is expected to be a valuable tool for exploring and analyzing AMR genes, the periodic updates of the tool will further establish it as a crucial resource for antibiotic resistance research.

## Data Availability

The BOARDS database is freely available at https://sbml.unist.ac.kr/ and can be accessed with a web server. Source code for the RADAR is freely available under an open-source license at https://github.com/SBML-Kimlab/radar.

## SUPPLEMENTAL MATERIAL

Supplemental figures msystems.00943-23-s0001.pdf Figures S1 to S19.

Table S1 msystems.00943-23-s0002.xlsx Selection criteria for consolidation into BOARDS.

Table S2 msystems.00943-23-s0003.xlsx The WGS analysis result of ESBL-associated AMR gene and non-ESBL MCR within 8 major antimicrobial-resistant bacteria.

Table S3 msystems.00943-23-s0004.xlsx Pan-resistome reconstruction of ESBL and non-ESBL MCR identified within eight major AMR bacteria based on WGS analysis.

Table S4 msystems.00943-23-s0005.xlsx The epidemiological investigation results of newly emerged AMR gene based on WGS analysis.

Table S5 msystems.00943-23-s0006.xlsx The result of co-occurrence analysis according to an epidemiological investigation of carbapenem and colistin resistance genes.

Table S6 msystems.00943-23-s0007.xlsx The results of atomic electrostatic characterization of each residue across four positions (104, 182, 238, 265) in a total of four TEM models.

Table S7 msystems.00943-23-s0008.xlsx The results of ligand ES environment modulation on the active site induced by point mutations.

Table S8 msystems.00943-23-s0009.xlsx The independent molecular docking simulation results between four TEM models and cefotaxime.

ASM does not own the copyrights to Supplemental Material that may be linked to, or accessed through, an article. The authors have granted ASM a non-exclusive, world-wide license to publish the Supplemental Material files. Please contact the corresponding author directly for reuse.
