# Supplementary material for: Blanket antimicrobial resistance gene database with structural information, BOARDS, provides insights on historical landscape of resistance prevalence and effects of mutations in enzyme structure
Source: mSystems. 2023 Dec 12;9(1):e00943-23. doi: 10.1128/msystems.00943-23 (PMC10871167; doi:10.1128/msystems.00943-23)
Supplement: Supplemental figures — Figures S1 to S19. [file msystems.00943-23-s0001.pdf]

Supplementary materials

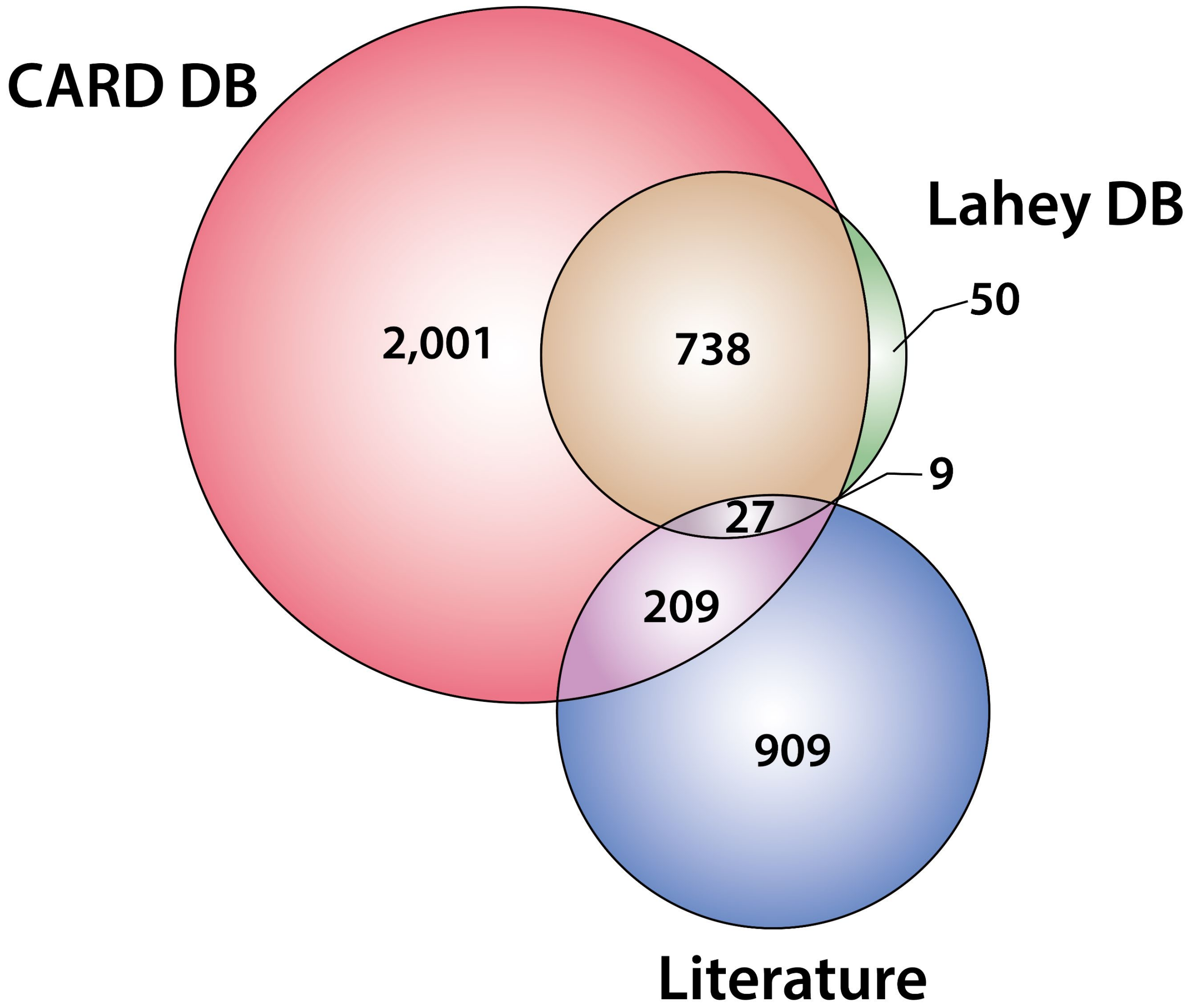

Supplementary Figure S1. The source composition of BOARDS consolidation. BOARDS consolidated antibiotic resistance gene information obtained from the CARD and Lahey clinic ESBL database, which are extant AMR gene databases, and literature review.

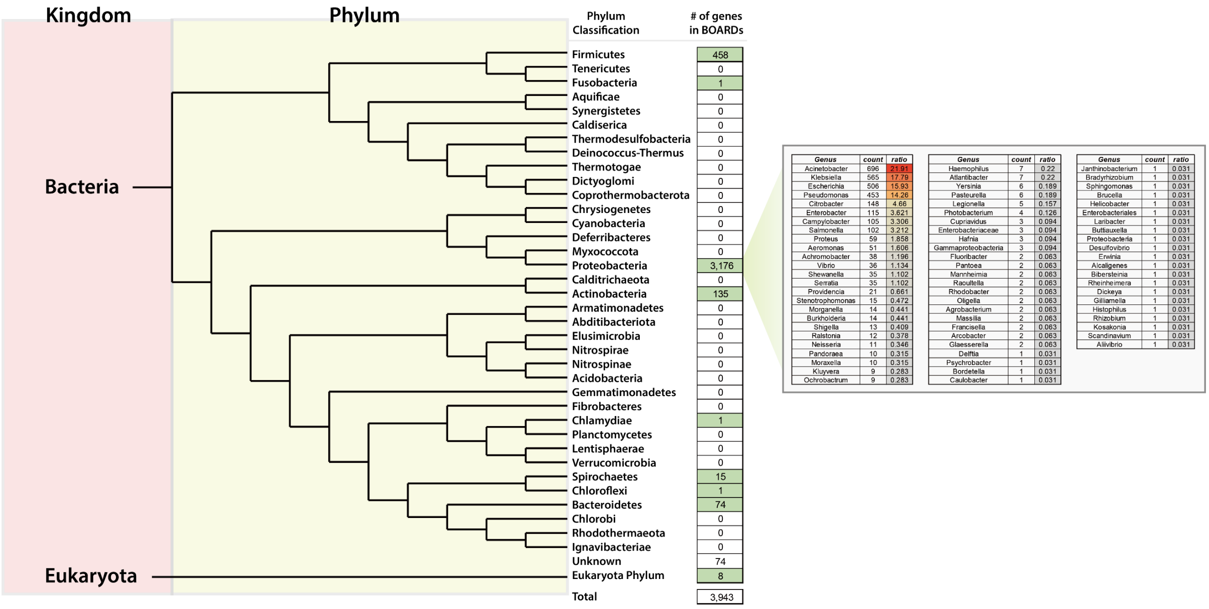

Supplementary Figure S2. Diversity of antimicrobial resistance genes possessed by the BOARDS.

The taxonomic classification by isolation source of all genes in BOARDS confirmed that the database contained the most genes belonging to *Proteobacteria*. In detail, it was confirmed that the most counted genus was *Acinetobacter*, which recorded 696. In addition, BOARDS contains other phyla such as *Firmicutes*, *Actinobacteria*, *Bacteroidetes*, *Spirochaetes*, phyla belonging to *Eukaryota*, *Fusobacteria*, *Chlamydiae*, and *Chloroflexi* as well.

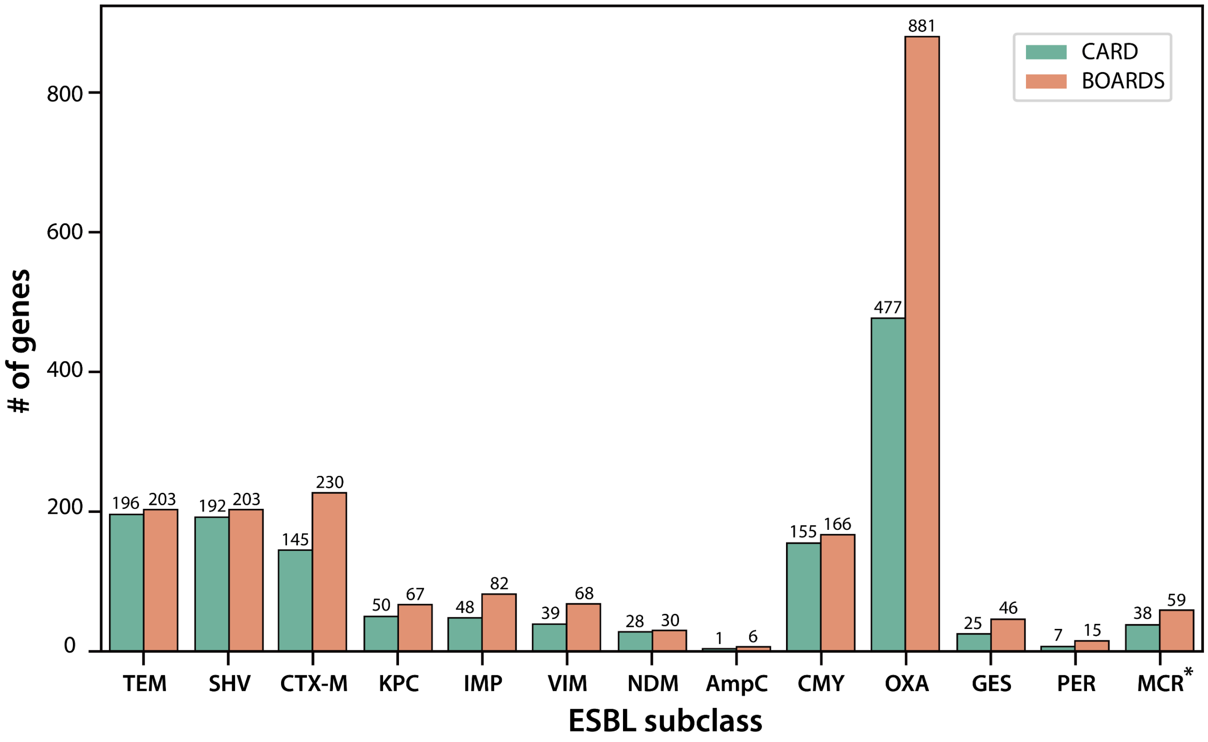

Supplementary Figure S3. The comparison of the number of ESBL antibiotic resistance genes in the BOARDS and CARD. It was found that BOARDS contained more ESBL genes than CARD (v3.2.1), according to Ambler classification. It was confirmed that the total ESBL subclass included in BOARDS was 1,997. Among them, the OXA subclass included 404 more than CARD. In addition, the MCR subclass, among non-ESBLs, was about 1.55 times more prevalent. The asterisk stands for non-ESBL.

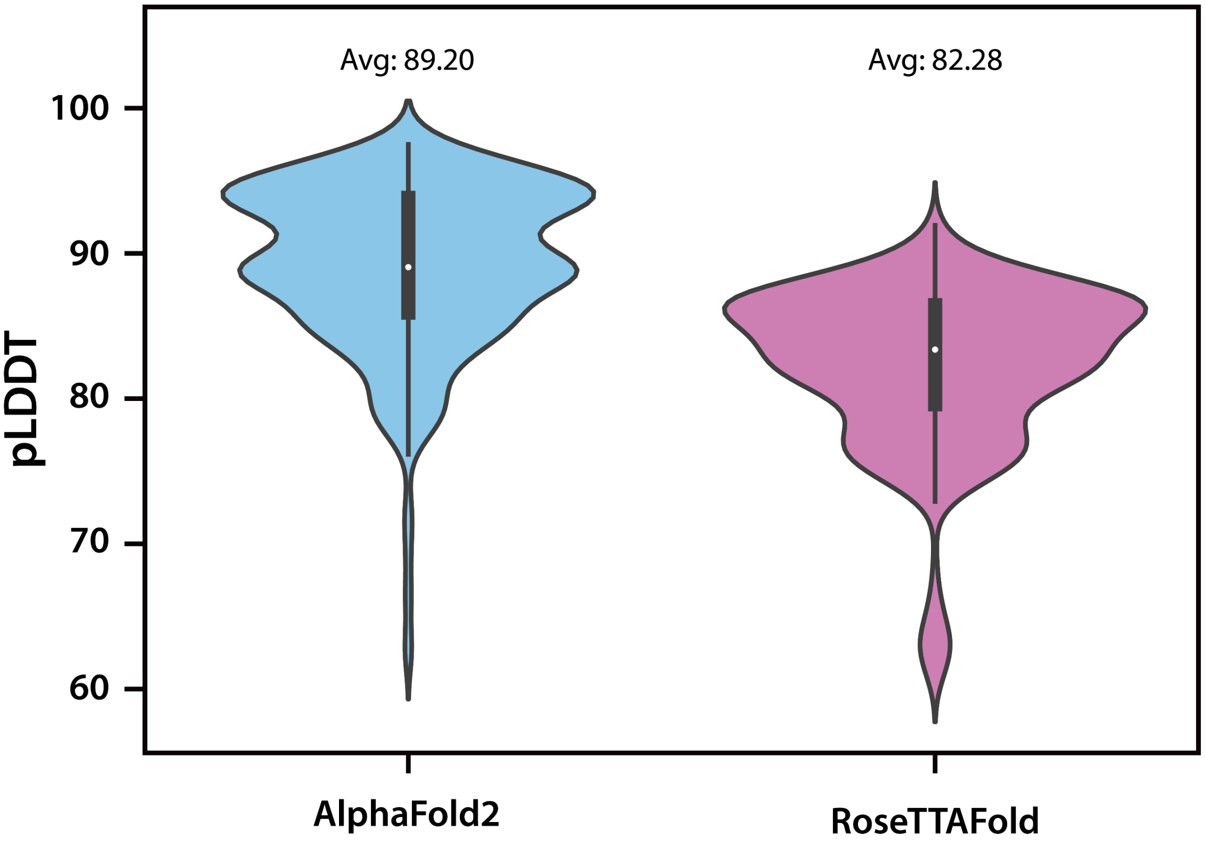

Supplementary Figure S4. The average pLDDT score comparison of predicted protein structural information of all the mutant models within BOARDS. The mutant models provided by BOARDS include the predicted protein structure using both AlphaFold2 and RoseTTAFold. A pLDDT score of 70 or higher is defined as a high-confidence predictive protein structure.

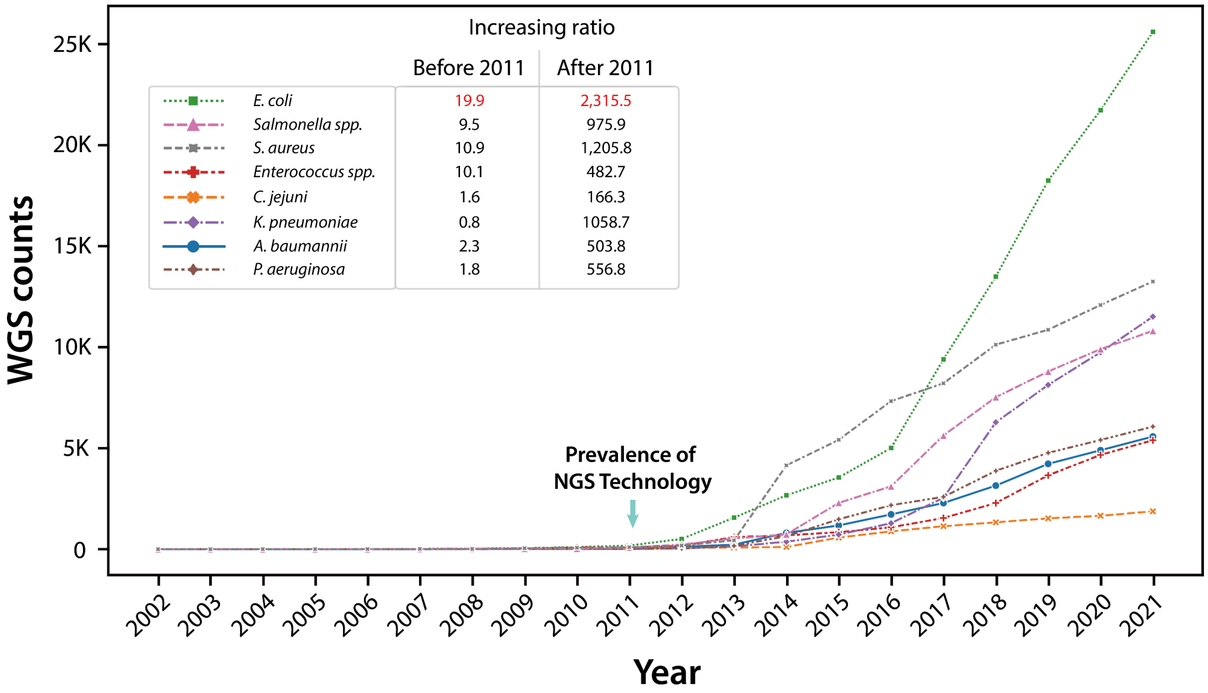

Supplementary Figure S5. The exponential increase in WGS data due to advances in sequencing technology. It was observed that the increase in WGS of a total of eight pathogens including ESKAPE pathogens increased exponentially. In particular, the increasing ratio of *E. coli* is 2315.5, which has been found to be the greatest increase in rate since the development of sequencing technology (2011).

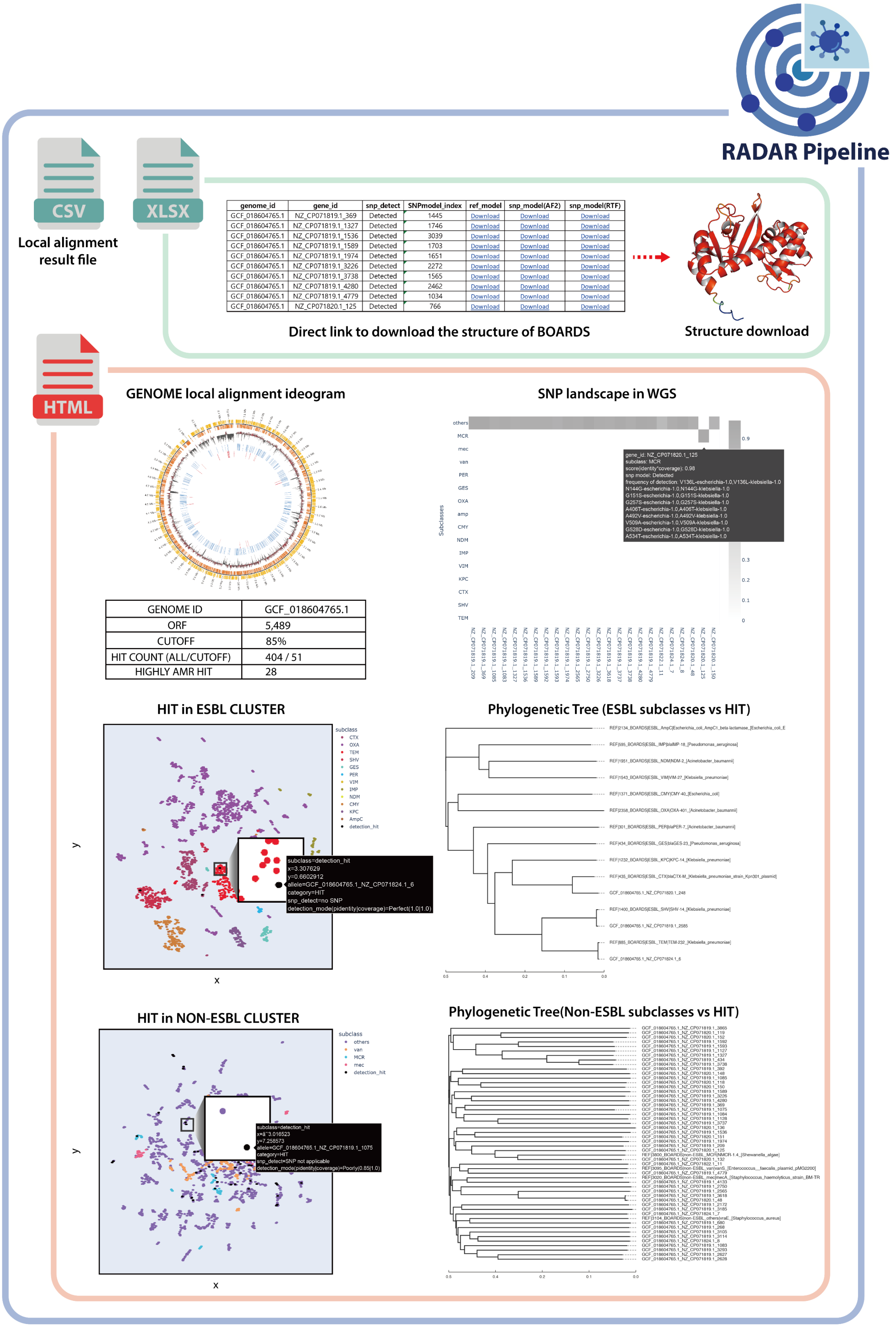

Supplementary Figure S6. Overview of the RADAR pipeline functionality. The RADAR pipeline processes WGS (Whole Genome Sequencing) data from users as input, analyzes it, and provides the

results in multiple formats. (A) The input WGS data is processed for local alignment against the BOARDS database, and the alignment results are provided in a tab-delimited csv format. (B) In case of detection of SNPs (Single Nucleotide Polymorphisms) in the AMR genes within the input WGS data that match frequently occurring SNP models in the BOARDS database, an xlsx file containing direct links to download the reference and mutant predictive protein structures is provided. (C) The results of the WGS data analyzed by RADAR are provided in an html format, comprising six distinct sections. The top-left section visualizes the local alignment results of the input WGS data in a table format and provides a circular representation of the genome alignment segments (Blue: hits for all AMR genes, Red: highly AMR hits that exceed the set cutoff threshold). The top-right section visualizes the distribution of the detected SNPs in the input data by ESBL subclass in the form of a heatmap. Hovering the mouse over the heatmap displays the probability of the detected SNP occurring in various hosts. The middle-left section visualizes the UMAP-based clustering results between all ESBL class AMR genes in BOARDS and the AMR hits detected in the input data. This section incorporates a zoom feature and allows users to click on figure legends to toggle the visibility of markers for each subclass. The middle-right section displays the phylogenetic tree between the representative sequences of each ESBL subclass and AMR hits. The bottom-left section visualizes the UMAP-based clustering results between all non-ESBL class AMR genes in BOARDS and the AMR hits detected in the input data. Same as the middle-left section, it also incorporates a zoom feature and enables marker visibility toggling for each subclass through the figure legend. The bottom-right section displays the phylogenetic results between the representative sequences of each non-ESBL subclass and AMR hits.

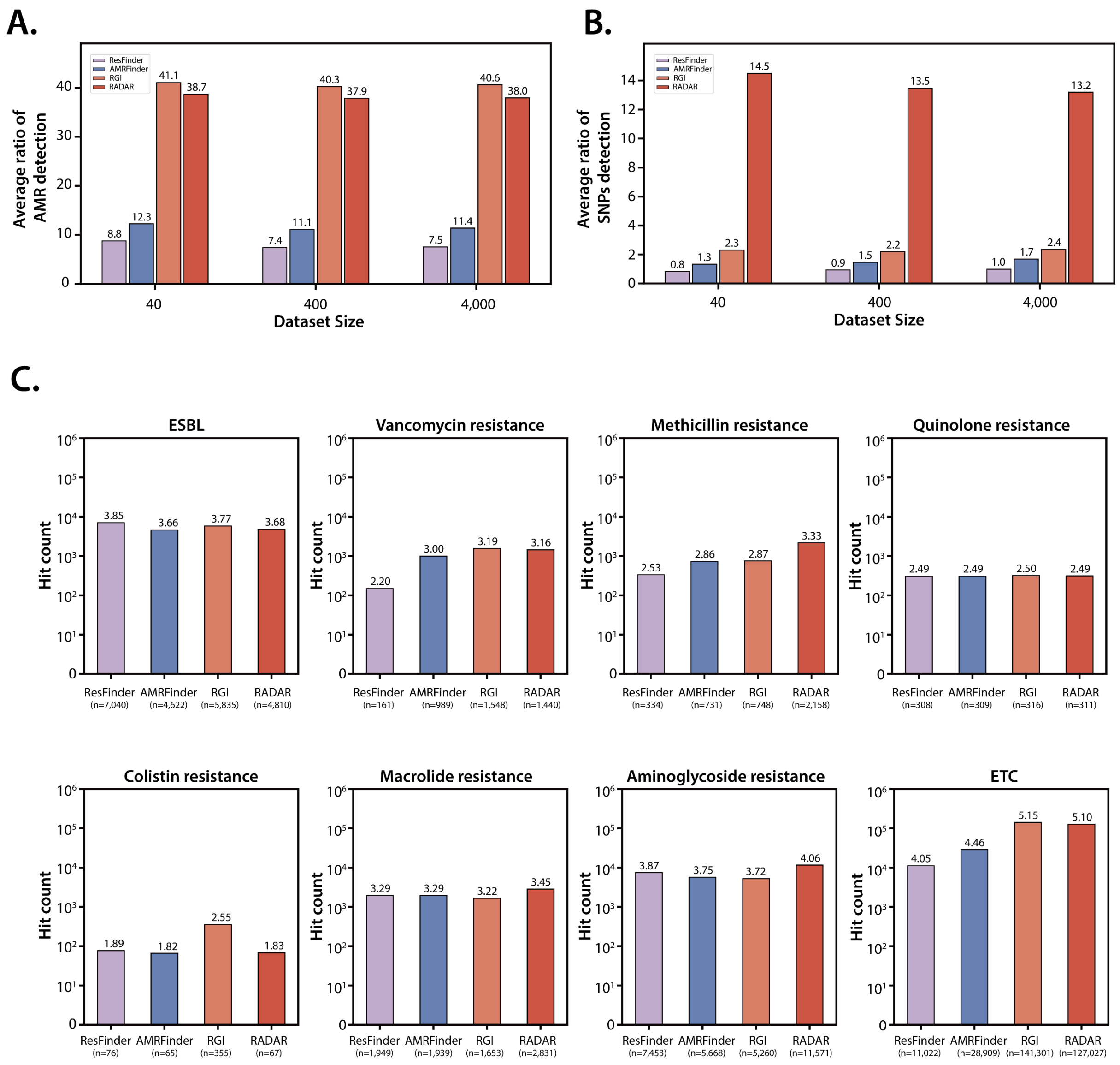

Supplementary Figure S7. Performance comparison of RADAR with previous AMR detection algorithms. (A) A comparison of AMR gene detection performance of the RADAR pipeline and previously existing AMR detection algorithms based on multiple dataset sizes (40, 400, 4,000 of WGS data) of major antibiotic-resistant pathogenic bacteria. The average number of AMR genes detected relative to the total genes by each AMR detection algorithm was displayed. The tools compared for detection performance were color-coded as shown in the legend. (B) A comparison of SNP detection performance of the RADAR pipeline and previously existing AMR detection algorithms based on multiple dataset sizes (40, 400, 4000 of WGS data). The average number of SNPs detected relative to the total genes by each AMR detection algorithm is represented. The tools compared for detection performance are differentiated by colors (ResFinder: purple, AMRFinder: blue, RGI: orange, RADAR:

red). (C) A comparison of AMR detection performance by each algorithm based on genes detected in each AMR gene class. For each AMR class, genes such as ESBL (TEM, CTX, SHV, KPC, IMP, VIM, NDM, AmpC, CMY, OXA, GES, PER), vancomycin resistance (*van*), methicillin resistance (*mec*), quinolone resistance (*qnr*), colistin resistance (*mcr*), macrolide resistance (*erm*, *mef*, *msr*, *mph*, *ere*), and aminoglycoside resistance (*aac*, *aad*, *aph*) have been visualized in terms of their quantity detected by each algorithm. The data values were plotted after being transformed using a log10 scale.

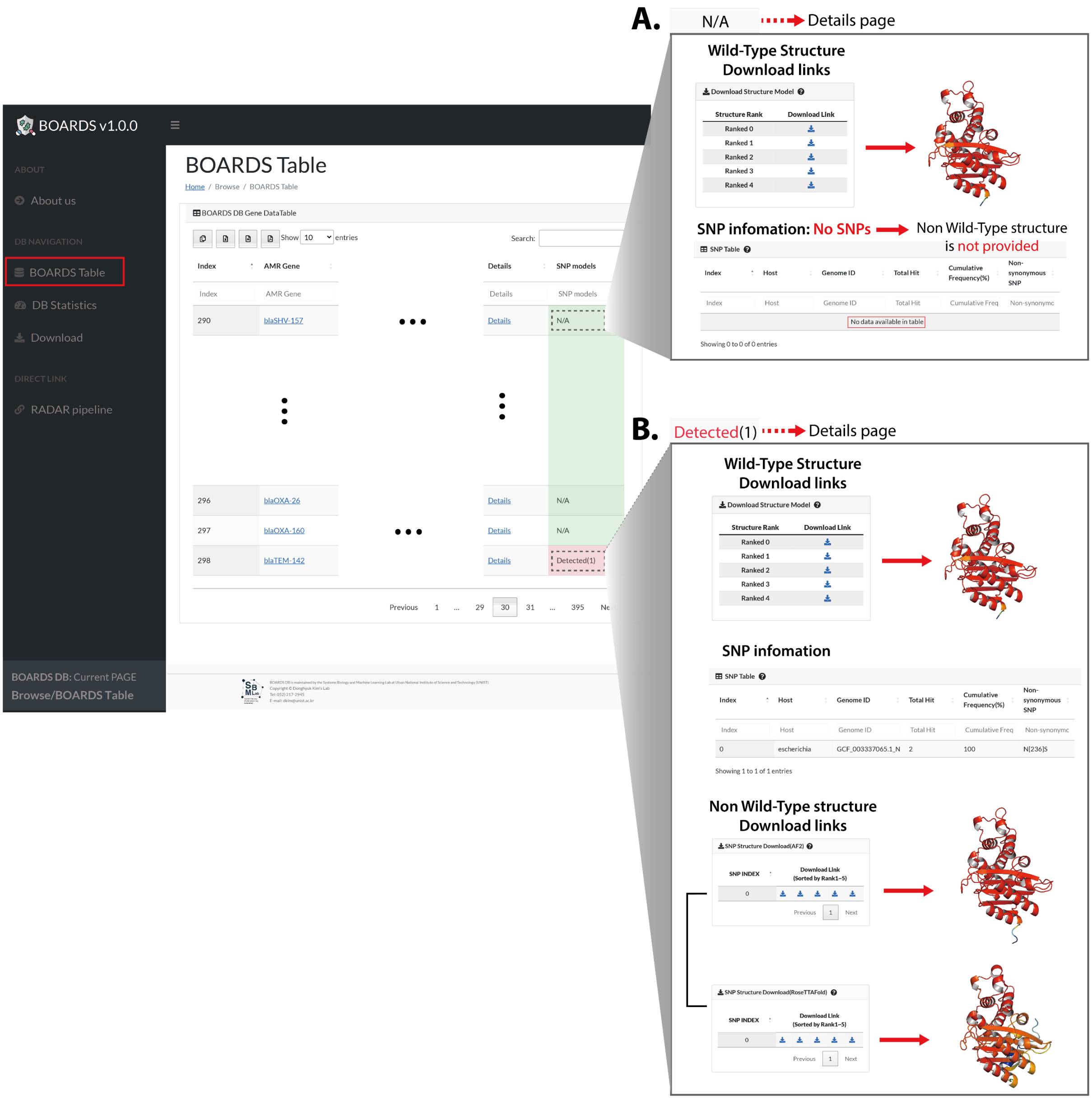

Supplementary Figure S8. Accessing the predicted protein structures on the BOARDS website. Within the BOARDS website, access is provided to the structures of antibiotic resistance genes and frequently occurring SNP models via the BOARDS Table. Displayed segments of the BOARDS Table list the AMR genes with specific identifiers and indices. For each entry, a "Details" link and the status of the existence of an "SNP model" are shown. (A) In the case where no SNP model is associated with a specific antibiotic resistance gene, "N/A" is indicated in the "SNP models" column. On the "Details" page, available wild-type protein structures and their respective download links for predicted protein structures are presented. If no SNPs are detected, no download link for the non-wild-type structure is provided. (B) In the case that an SNP model detects for a particular gene, "Detected" is indicated in the

"SNP models" column, with the number in parentheses representing the number of detected SNPs. On the "Details" page for that AMR gene entry, the available wild-type protein structures and their download links are displayed, followed by activated download links for the non-wild type structures. Notably, each index in the SNP table activates the download link for its respective non-wild-type structure, allowing users to access structures related to specific SNPs.

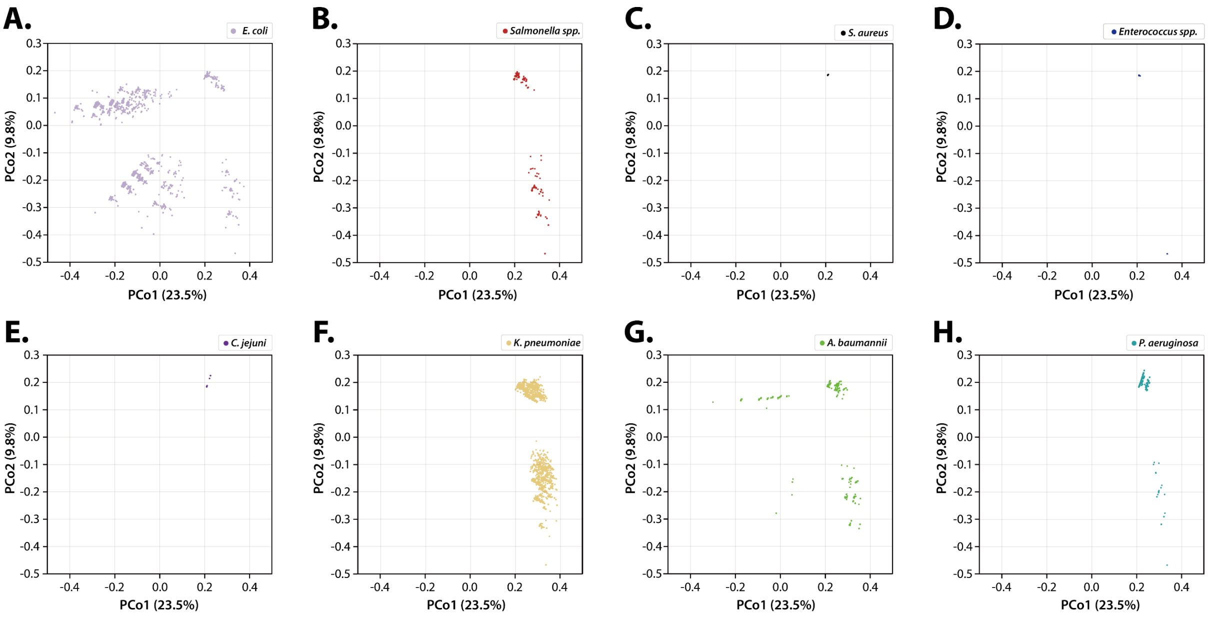

Supplementary Figure S9. Pan-resistome reconstruction of eight pathogens: distribution patterns of the AMR genes according to species. (A) The pan-resistome reconstructed according to the ESBL gene distribution showed that *E. coli* was globally arranged. (B) The pattern of *Salmonella* spp. was not globally distributed, and it was confirmed that it showed a biased distribution pattern. (C) Although *S. aureus* had only 18 hits in total, it was observed that all hits had a similar distribution in a dense pattern. (D) In *Enterococcus* spp., although the distribution pattern of one hit was different, it was observed that overall distribution was similar to that of *S. aureus*. (E) *C. jejuni* was observed to have a dense distribution pattern with more than 1000 hits. (F) A biased distribution pattern was observed in *K. pneumoniae* similar to that of *Salmonella* spp. (G) It was confirmed that the distribution pattern of *A. baumannii* was globally arranged. (H) *P. aeruginosa* showed a biased distribution pattern similar to that of *Salmonalla* spp. and *K. pneumoniae*.

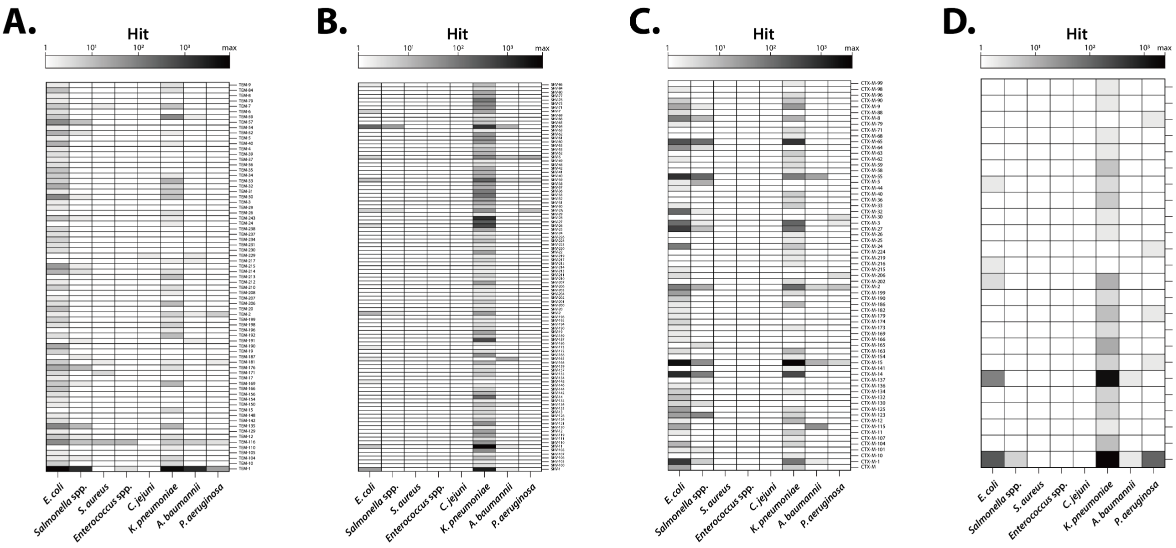

Supplementary Figure S10. Pan-resistome reconstruction of eight pathogens (Class A): distribution patterns of the AMR genes according to subclasses in ESBL. The heatmap colors represent log10 transformed data of subclass hits. (A) Distribution patterns of antibiotic resistance genes corresponding to TEM subclasses. The TEM subclass was detected in all eight pathogens, and it was confirmed that the largest number was detected in *E. coli*. There was a total of 73 genes corresponding to the detected TEM subclass, and it was identified that the most detected gene was TEM-1. (B) Distribution pattern of antibiotic resistance genes corresponding to the SHV subclass. The SHV subclass was found in all other pathogens except *S. aureus*, *Enterococcus* spp., and *C. jejuni*. It was confirmed that the largest number was detected in *K. pneumoniae*. There was a total of 102 genes corresponding to the detected SHV subclass, and it was identified that the most detected gene was SHV-11. (C) Distribution pattern of antibiotic resistance genes corresponding to the CTX-M subclass. The CTX-M subclass was found in all pathogens except *S. aureus*, *Enterococcus* spp., and *C. jejuni*. It was confirmed that the largest number was detected in *E. coli*. There was a total of 67 genes corresponding to the detected CTX-M subclass, and it was identified that the most detected gene was CTX-M-15. (D) Distribution pattern of antibiotic resistance gene corresponding to KPC subclass. The KPC subclass was found in all pathogens except *S. aureus*, *Enterococcus* spp., and *C. jejuni*. It was confirmed that the largest number was detected in *K. pneumoniae*. There was a total of 24 genes corresponding to the detected KPC subclass, and it was identified that the most detected gene was KPC-1.

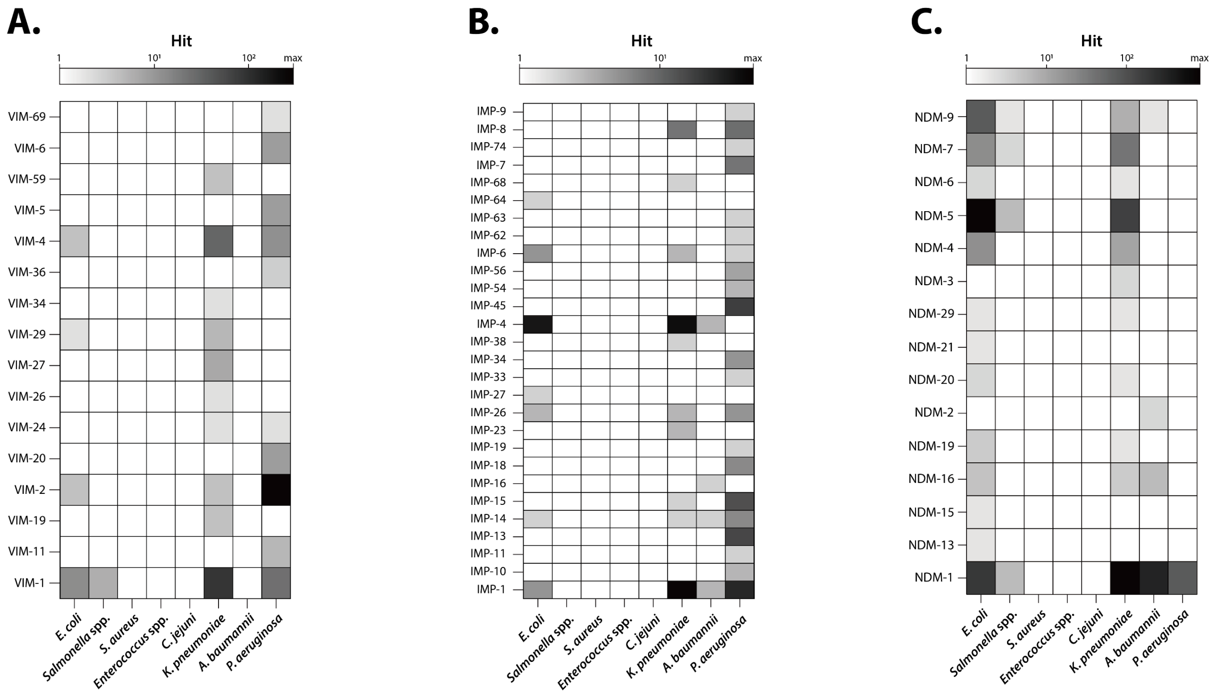

Supplementary Figure S11. Pan-resistome reconstruction of eight pathogens (Class B): distribution patterns of the AMR genes according to subclasses in ESBL. The heatmap colors represent log10 transformed data of subclass hits. (A) Distribution patterns of AMR genes corresponding to VIM subclasses. The VIM subclass was found in *E. coli*, *Salmonella* spp., *K. pneumoniae*, and *P. aeruginosa*. It was confirmed that the largest number was detected in *P. aeruginosa*. There was a total of 16 genes corresponding to the detected VIM subclass, and it was identified that the most detected gene was VIM-2. (B) Distribution patterns of AMR genes corresponding to IMP subclasses. The IMP subclass was found in *E. coli*, *K. pneumoniae*, *A. baumannii*, and *P. aeruginosa*. It was confirmed that the largest number was detected in *P. aeruginosa*. There was a total of 28 genes corresponding to the detected IMP subclass, and it was identified that the most detected gene was IMP-1. (C) Distribution patterns of AMR genes corresponding to NDM subclasses. The NDM subclass was found in all other pathogens except *S. aureus*, *Enterococcus* spp., and *C. jejuni*. It was confirmed that the largest number was detected in *E. coli*. There was a total of 15 genes corresponding to the detected NDM subclass, and it was identified that the most detected gene was NDM-1.

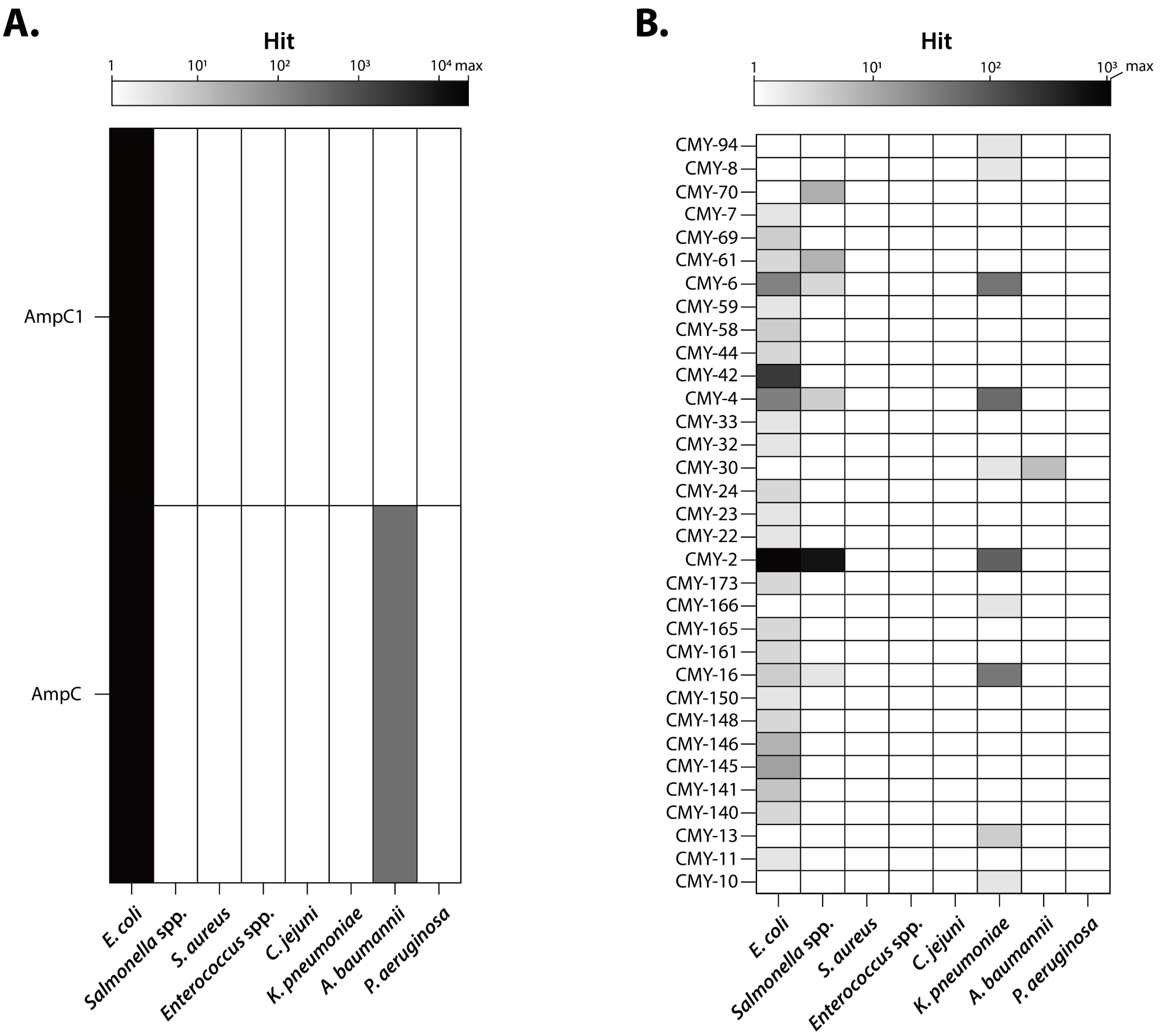

Supplementary Figure S12. Pan-resistome reconstruction of eight pathogens (Class C): distribution patterns of the AMR genes according to subclasses in ESBL. The heatmap colors represent log10 transformed data of subclass hits. (A) Distribution patterns of AMR genes corresponding to ampC subclasses. The ampC subclass was found in only *E. coli* and *A. baumannii*. It was confirmed that the largest number was detected in *E. coli*. There was a total of 2 genes corresponding to the detected ampC subclass, and it was identified that the most detected gene was ampC. (B) Distribution patterns of AMR genes corresponding to CMY subclasses. The CMY subclass was found in all other pathogens except *S. aureus*, *Enterococcus* spp., *C. jejuni*, and *P. aeruginosa*. It was confirmed that the largest number was detected in *E. coli*. There was a total of 33 genes corresponding to the detected CMY subclass, and it was identified that the most detected gene was CMY-2.

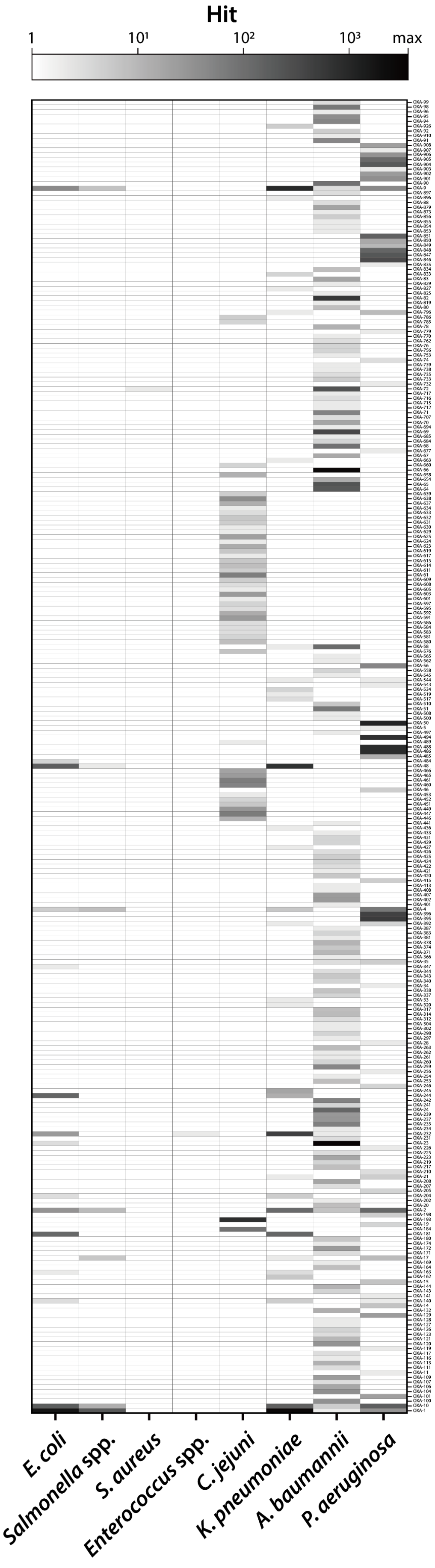
Hit
1
10¹
10²
10³
max
E. coli
Salmonella spp.
S. aureus
Enterococcus spp.
C. jejuni
K. pneumoniae
A. baumannii
P. aeruginosa

Supplementary Figure S13. Pan-resistome reconstruction of eight pathogens (Class D): distribution patterns of the AMR genes according to subclasses in ESBL. The heatmap colors represent log10 transformed data of subclass hits. Distribution patterns of AMR genes corresponding to OXA subclasses. The OXA subclass was detected in all eight pathogens, and it was confirmed that the largest number was detected in *A. baumannii*. There was a total of 275 genes corresponding to the detected OXA subclass, and it was identified that the most detected gene was OXA-1.

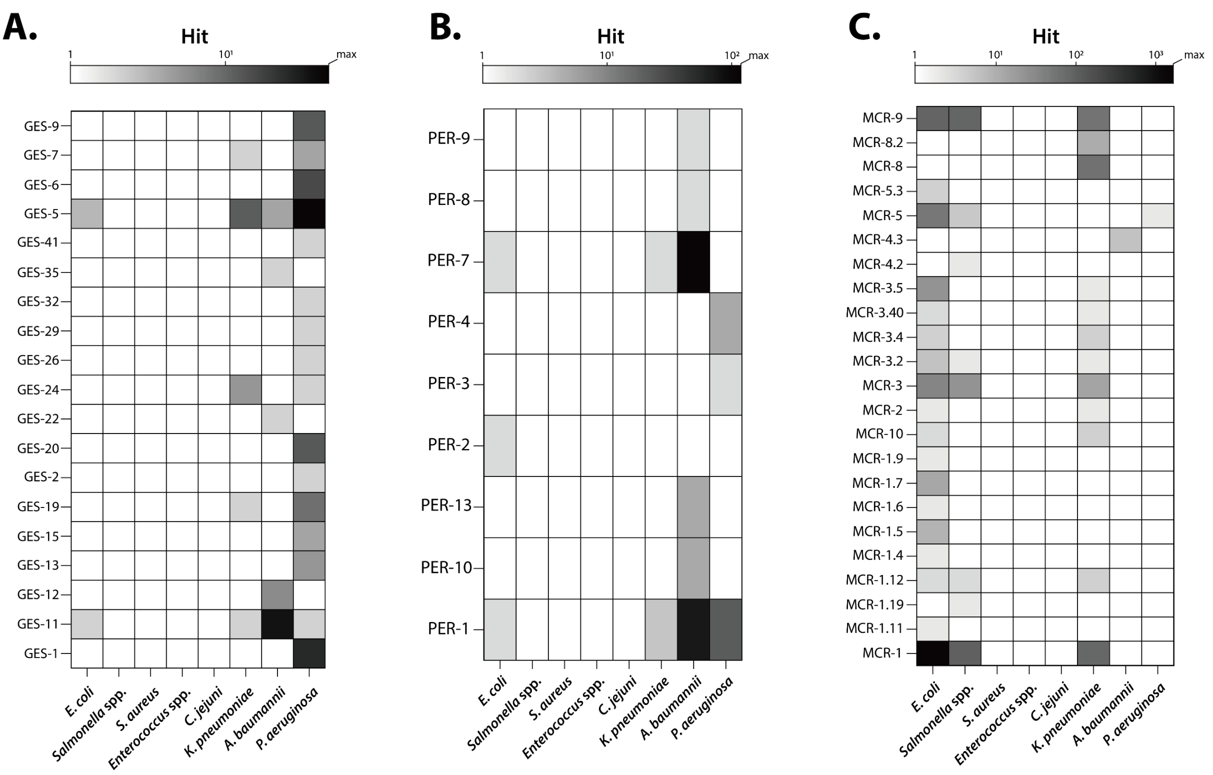

Supplementary Figure S14. Pan-resistome reconstruction of eight pathogens (Miscellaneous ESBL subclass and MCR of Non-ESBL): distribution patterns of the AMR genes according to subclasses of ESBL not classified according to Ambler Classification and the Non-ESBL, MCR. The heatmap colors represent log10 transformed data of subclass hits. (A) Distribution patterns of miscellaneous AMR genes corresponding to GES subclasses which are not classified according to Ambler Classification. The GES subclass was found in *E. coli*, *K. pneumoniae*, *A. baumannii*, and *P. aeruginosa*. It was confirmed that the largest number was detected in *P. aeruginosa*. There was a total of 19 genes corresponding to the detected GES subclass, and it was identified that the most detected gene was GES-5. (B) Distribution patterns of AMR genes corresponding to PER subclasses, which are miscellaneous subclasses of ESBL. The PER subclass was found in *E. coli*, *K. pneumoniae*, *A. baumannii*, and *P. aeruginosa*. It was confirmed that the largest number was detected in *A. baumannii*. There was a total of 9 genes corresponding to the detected PER subclass, and it was identified that the most detected gene was PER-7. (C) Distribution patterns of AMR genes corresponding to the MCR subclass, which is categorized as Non-ESBL. The MCR subclass was found in all pathogens except *S. aureus*, *Enterococcus* spp., and *C. jejuni*. It was confirmed that the largest number was detected in *E.*

coli. There was a total of 23 genes corresponding to the detected MCR subclass, and it was identified that the most detected gene was MCR-1.

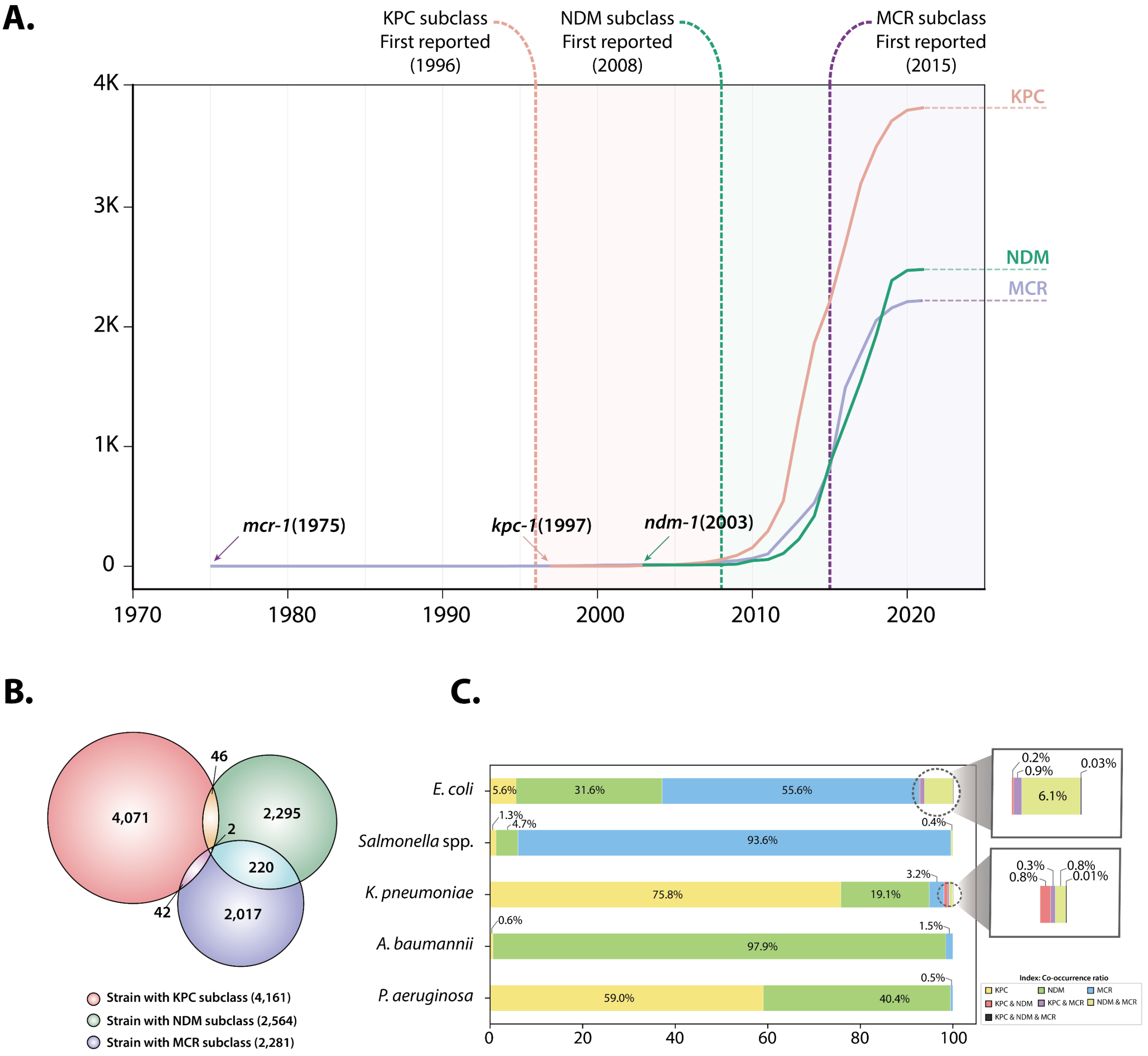

Supplementary Figure S15. The epidemiology analysis results for KPC, NDM, and MCR which were newly-emerged AMR subclasses. (A) Year-by-year increase graph of three newly emerged AMR genes identified through epidemiological investigation based on WGS analysis. The three newly emerged AMR genes are gradually increasing, and it has been found that NDM and MCR existed before they were first reported. It was shown that the first isolated MCR hit was isolated in 1975. (B) Coexistence of hits of three newly emerged AMR genes obtained in this study through WGS analysis. (C) The co-existence ratio of three AMR subclasses (KPC, NDM, and MCR) in a total of five strains in

which newly emerged AMR genes were detected.

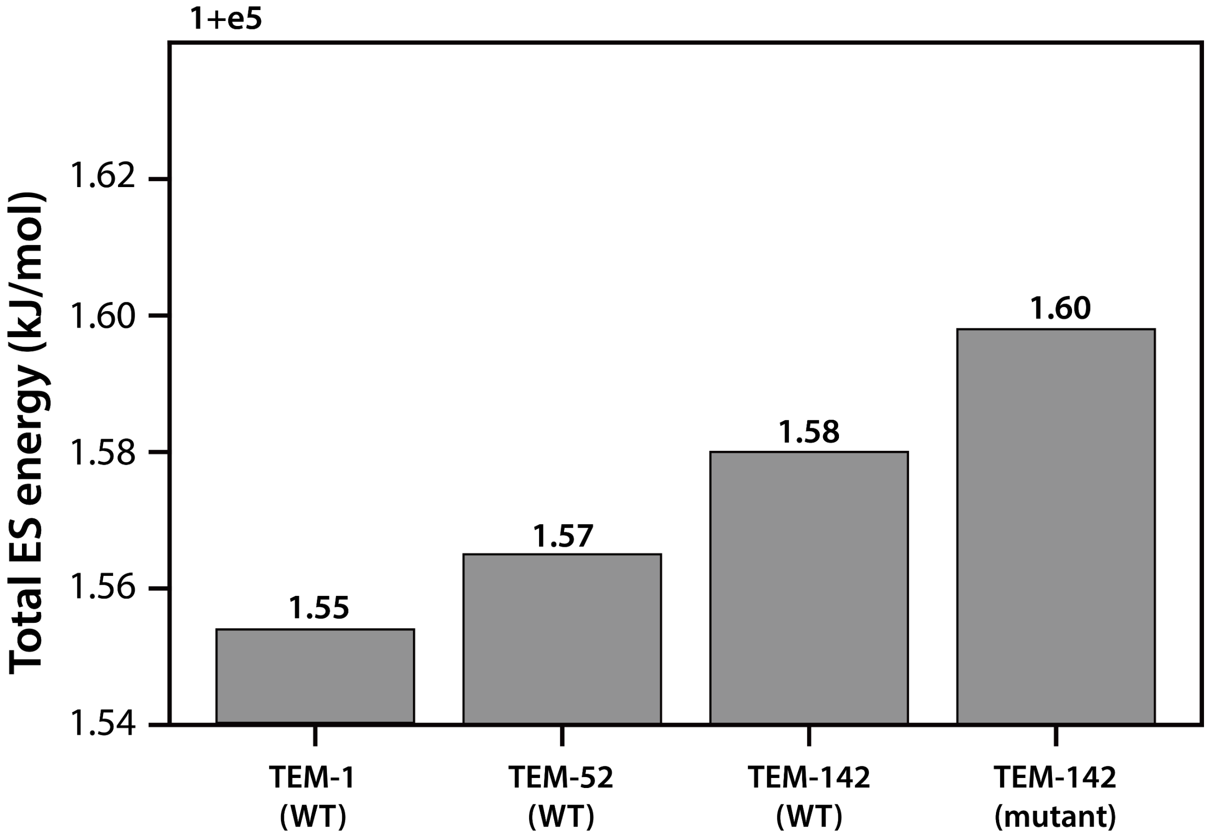

Supplementary Figure S16. The total electrostatic energy of four TEM subclass genes predicted protein structures provided by the BOARDS. As for the total ES energy, it was confirmed that TEM-52, with multiple point mutations, had higher ES energy than TEM-1 (WT). Moreover, a higher total ES energy was observed in the TEM-142 (mutant) model caused by point mutation of TEM-142 (WT) followed the same trend.

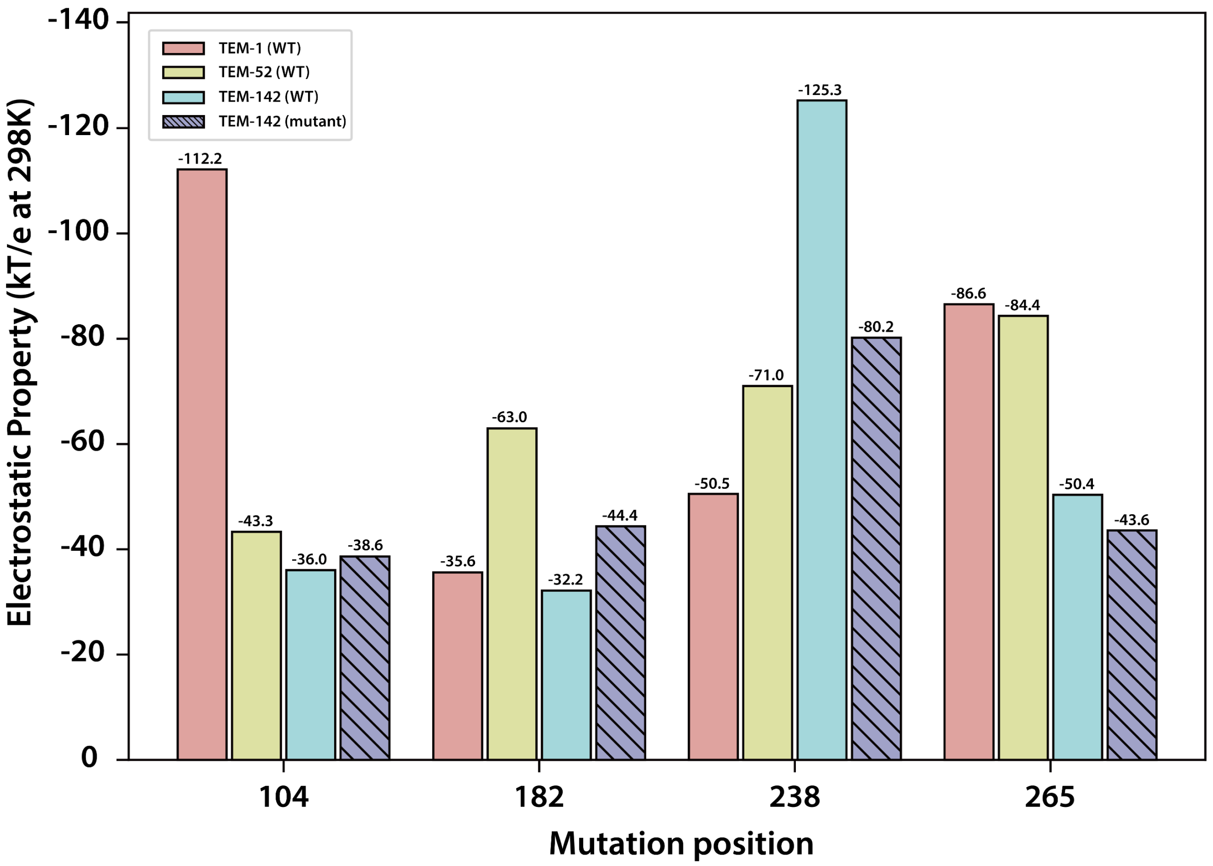

Supplementary Figure S17. The difference in ES potentials in key residues in four positions where mutations occur. The indicated score is the average value of the ES potentials score of all atoms constituting the residue at each position. The ES potentials score of position 238, where the most substitution occurs, shows the largest change in value when glycine is substituted with asparagine. Additionally, it was shown that position 104 has the greatest ES differentials when substitution occurs.

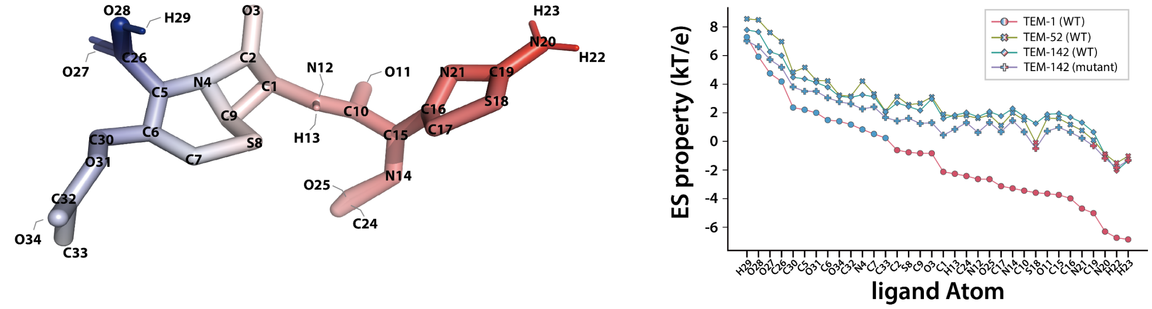

Supplementary Figure S18. For each of the four TEM models provided by BOARDS, the ES property of each atom of cefotaxime, which is a substrate, is displayed. (A) Each atom number assigned to the substrate, cefotaxime. (B) It was confirmed that TEM-1 and the other three models had different trends for the ES potentials of each atom constituting cefotaxime. Blue indicates positive ES potential, and red indicates negative ES potential.

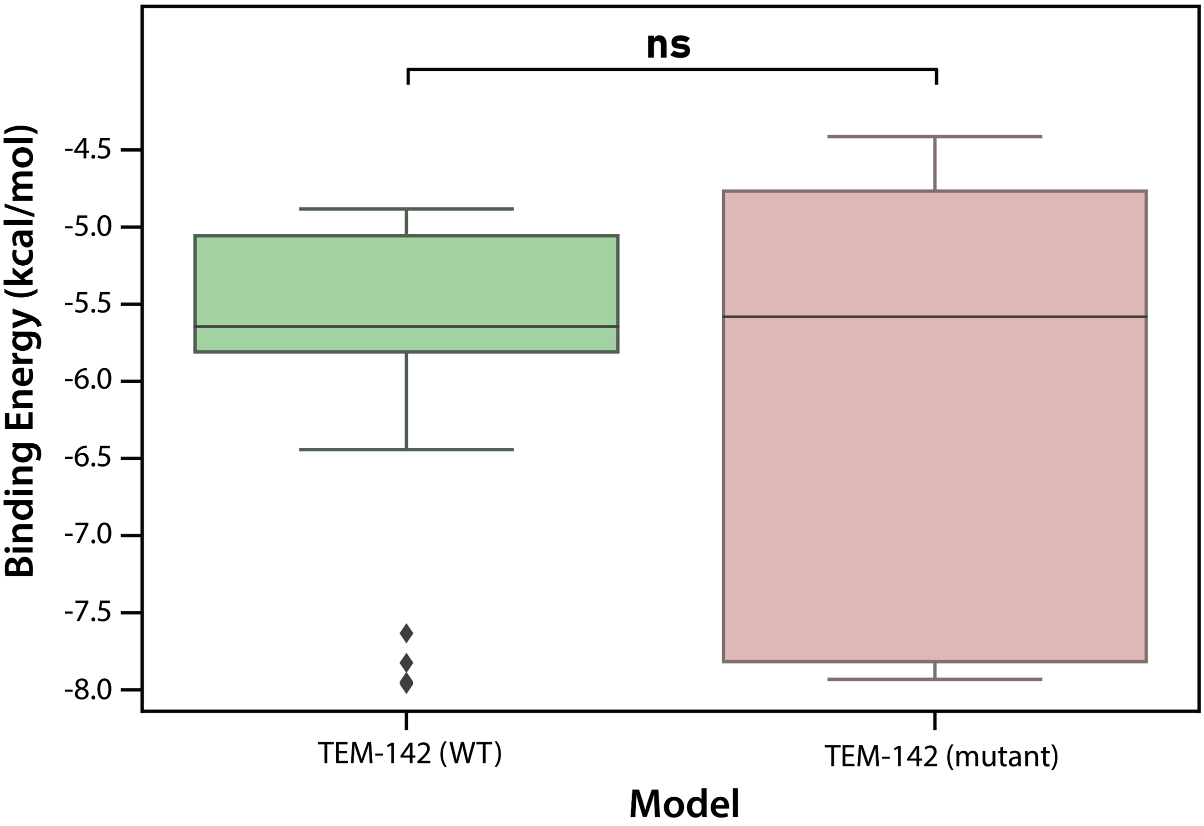

Supplementary Figure S19. The docking simulation result of the mutant model provided by BOARDS. The mutant model was provided by BOARDS and the Wild-Type model was additionally predicted through RoseTTAFold to compare molecular docking simulation results. It was shown that the TEM-142 (mutant) model in which the TEM-142 (WT) was substituted had low binding energy, but it was not statistically significant.
